# Selection, Comparative Genomics, and Potential Probiotic Features of *Escherichia coli* 5C, a *pks*-Negative Strain Isolated from Healthy Infant Donor Feces

**DOI:** 10.1007/s12602-025-10522-5

**Published:** 2025-04-16

**Authors:** Francesco Di Pierro, Valeria Sagheddu, Serena Galletti, Alice Casaroli, Edoardo Labrini, Sara Soldi, Massimiliano Cazzaniga, Alexander Bertuccioli, Mariarosaria Matera, Ilaria Cavecchia, Chiara Maria Palazzi, Maria Laura Tanda, Nicola Zerbinati

**Affiliations:** 1 Microbiota International Clinical Society, 10123 Turin, Italy; 2 Scientific & Research Department, Velleja Research, 20125 Milan, Italy; 3 Department of Medicine and Technological Innovation, University of Insubria, 21100 Varese, Italy; 4 AAT-Advanced Analytical Technologies, Fiorenzuola d’Arda, 29017 Piacenza, Italy; 5 Department of Biomolecular Sciences, University of Urbino Carlo Bo, 61122 Urbino, Italy; 6 Department of Pediatric Emergencies, Misericordia Hospital, 58100 Grosseto, Italy; 7 Microbiomic Department, Koelliker Hospital, 10134 Turin, Italy; 8 Endocrine Unit, Department of Medicine and Surgery, University of Insubria, 21100 Varese, Italy

**Keywords:** LMG S-33222 (5C), ECP24®, Probiotics, Colibactin, Virulence factors, Mutagenic

## Abstract

Among the emerging issues in probiotic safety, the possible presence of *pks*, a gene cluster synthetizing a genotoxin known as colibactin, is one of the most alarming. Indeed, indigenous *E. coli* strain *pks*-positive are found in 60% of patients with colorectal cancer, and the most widely used *E. coli*-based probiotic, known as *E. coli* Nissle 1917 (DSM 6601), is *pks*-positive. Starting from 25 potential candidates selected by screening 25 infant stool samples, we have selected an *E. coli* strain (named 5C, deposited as LMG S-33222) belonging to the phylotype A and having the serovar O173:H1. Having been previously completely sequenced by our group, we have further characterized this strain, demonstrating that it is (i) devoid of the most known potential pathogenic-related genes, (ii) devoid of possible plasmids, (iii) antibiotic-sensitive according to the EFSA panel, (iv) resistant in gastric and enteric juice, (v) significantly producing acetate, (vi) poorly producing histamine, (vii) endowed with a significant in vitro antipathogenic profile, (viii) promoting a significant in vitro immunological response based on IL-10 and IL-12, and (ix) devoid of the *pks* genes. A comparative genomics versus *E. coli* Nissle 1917 is also provided. Considering that the other two most commonly used *E. coli*-based probiotics (*E. coli* DSM 17252 and *E. coli* A0 34/86) are respectively *pks*-positive and alpha-hemolysin-(*hly*) and cytotoxic necrotizing factor-1-(*cnf1*) positive, this novel strain (*E. coli* 5C) is likely the probiotic *E. coli* strain with the best safety profile available to date for human use.

**Supplementary Information:**

The online version contains supplementary material available at 10.1007/s12602-025-10522-5.

## Introduction

### The Concept of Safety for Probiotics

The gut microbiota plays a central role in maintaining human health and influences digestive, immune, metabolic, and neurological functions, with probable repercussions also in the oncological field [1–3]. Understanding what affects its composition and diversity and promoting its eubiosis is therefore fundamental [4]. Numerous studies have demonstrated the beneficial effects deriving from the use of probiotics, most frequently attributable to the *Bifidobacterium* and *Lactobacillus* species, on human health [5–7]. Although there is consensus on the safety of using known and extensively tested probiotics [8–10], potential adverse events may occur when they are administered to immunocompromised individuals or to subjects with weakened immune systems, such as pediatric patients, transplant recipients, or patients undergoing chemotherapy [11–14]. Sometimes, intestinal discomfort may also occur, which is generally transient and decreases as the microbiota adapts [15]. More rarely, the administration of probiotics may (i) lead to hypersensitivity reactions (skin rashes, itching, and swelling); (ii) alter the effects of treatments by interfering with their efficacy; (iii) be involved in the production and metabolism of histamine (potentially promoting intestinal inflammatory response); or (iv) be associated with the transmission of antibiotic resistance elements or with the production of genotoxic metabolites, such as colibactin [16–23].

### The Probiotic Strain *E. coli* Nissle 1917 and the Cancer-Colibactin Relationship

*E. coli* Nissle 1917 is a Gram-negative member of the B2 phylogenetic group of *Escherichia coli*, isolated by Alfred Nissle in 1917 from the stool of a German soldier. Deployed in the Dobruja region for some time, then heavily contaminated by *Shigella*, this soldier, unlike his comrades, did not develop diarrhea or other intestinal diseases. Dr. A. Nissle hypothesized that he was a carrier of a strain of *E. coli* capable of direct antagonism towards other possible pathogenic enterobacteria. Indeed, he isolated from a stool sample of him a strain of *E. coli* that, in laboratory tests, demonstrated antagonistic activity towards other intestinal pathogens [24, 25]. Since then, the strain was formulated as a probiotic supplement and commercialized in Germany (Mutaflor®, Ardeypharm; Germany) and, later, in many other European countries (i. e. EcN®, Cadigroup, Italy). Currently, the strain, hereafter called *E. coli* Nissle 1917, or simpler Nissle 1917 or EcN, is deposited at the German Collection for Microorganisms and Cell Cultures (Deutsche Sammlung von Mikroorganismen und Zellkulturen, DSMZ), where it has got the designation *E. coli* DSM 6601 [25]. *E. coli* Nissle 1917’s basic microbiological and molecular features include (i) having common type fimbria (F1A), F1C fimbria, and Curli fimbria (respectively described as involved in biofilm formation and adhesion (F1A), gut and bladder colonization (F1C), and in bacterial aggregation (Curli) [26, 27]; (ii) having siderophores for iron acquisition (anti-inflammatory and/or fitness factors) [26, 28]; (iii) having O6 repeating units for LPS (a feature commonly found in extra-intestinal pathogenic *E. coli* strains), K5-type for capsule (described to make serum-resistant the extra-intestinal pathogenic *E. coli* strains), and H1-type for flagella (responsible for strain motility) [26, 29, 30]; (iv) having two plasmids (pMUT1 and pMUT2) synthetizing microcins M and H47 (active against *Salmonella* strains) [26, 31, 32]; (v) being strong bile-resistant and colonizing also due to the gene *Rfah* [26, 33]; and (vi) having the capability of synthetizing and releasing colibactin, a mutagenic substance described to promote colorectal polyposis and cancer, and reducing cancer response to irinotecan [26, 34–39]. As regards the cancer–colibactin relationship, very recent metagenomics-based and population-genomics-based surveys have shown that the prevalence of dominant colibactin-producing lineages of *E. coli* varies considerably across geographical regions, being higher in countries with a high human development index (HDI) like UK and USA and lower in countries with low HDI, like Pakistan and Bangladesh. This prevalence is strongly associated with the age-standardized incidences of colorectal cancer, bladder cancer, and prostate cancer, suggesting that the degree of colibactin exposure in a population might contribute to the geographical variation of these cancers [40]. Although some of these characteristics reported above from (i) to (vi) may be considered negative for a probiotic, the strain is widely used, especially (i) in the management of patients diagnosed with ulcerative colitis, but in remission, (ii) in childhood gastroenteritis, and (iii) in adult constipation [26, 41–44].

### Other *E. coli* Probiotic Strains

*E. coli* Nissle 1917 is not the only *E. coli* probiotic currently clinically used. A mixture of *E. coli* strains, (named G1/2, G3/10, G4/9, G5, G6/7, G8) deposited as DSM 17252 and commercially available as Symbioflor® 2 (SymbioPharm, Germany) is used for treating IBS. Moreover, the strain *E. coli* A0 34/86, commercially available as Colinfant® New Born (Dyntec, Czech Republic), is mainly used in newborns for preventing infections and atopy [45, 46]. Noteworthy, *E. coli* A0 34/86, a strain apparently devoid of the *pks* gene [47], contains at least two potential pathogenic genes (*hly*, *cnf-1*) respectively responsible for alpha-hemolysin and cytotoxic necrotizing factor-1 production and release [48]. As regards the *E. coli* mixture described before (DSM 17252), it is devoid of potential pathogenic genes, but the information about the gene *pks* is not precisely reported [49]. To our knowledge, only the strain *E. coli* CEC15, isolated from a suckling rat pup and initially developed for probiotic purpose, but never commercialized for human use, is a *pks*-negative strain [50]. The strain is positive for the *hly* gene [50]. Starting from these assumptions, we attempted to isolate from infant stool a strain of *E. coli* that, despite the expression of those fitness factors that characterize this species, presented a safety profile considered acceptable for a probiotic, including the complete absence of the *pks* gene. We have then made a genome comparison between this newly selected strain and *E. coli* Nissle 1917. Finally, since we were unable to establish, at least on a bibliographical basis, whether the probiotic registered as DSM 17252 contains the *pks* gene or not, we searched for it via PCR analysis directly in the finished product (Symbioflor® 2).

## Materials and Methods

### Bacterial *E. coli* Isolation from Human Feces

**Fig. 1:**
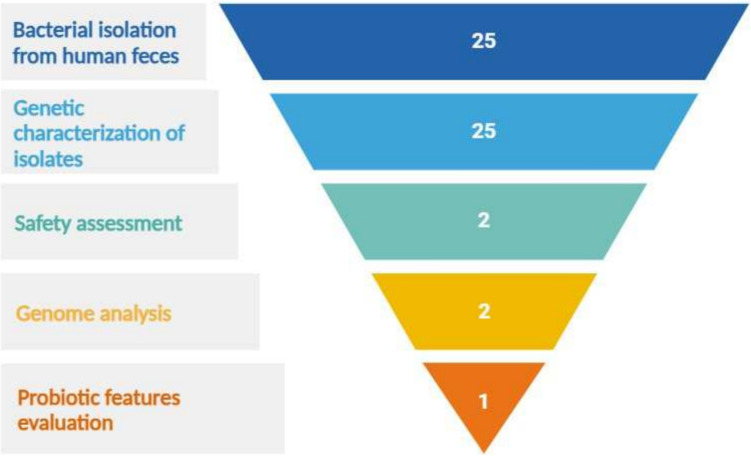
The funnel-like approach used to identify new putative probiotic strains

Fecal samples from 11 healthy children aged between 3 months and 4 years were freshly collected and plated onto TBX plates (Merck, Milan, Italy) and incubated for 24 h at 37 °C under aerobic conditions. Blue colonies, presumably belonging to *E. coli* species, were isolated and plated for three subculturing steps on TBX agar to ensure their purity. Twenty-five newly isolated strains of *E. coli* were recovered from the isolation step. The funnel-like approach, presented in Fig. 1, was followed to identify putative new probiotic strains.

### Species-Specific Identification of New Isolates

*E. coli* blue colonies grown on TBX agar were lysed for DNA extraction using the Whatman™ CloneSaver™ Card System (96-well format) (VWR, Milan, Italy). DNA extraction was performed according to the manufacturer’s instructions. Firstly, the identification of *E. coli* species was performed by a specific PCR protocol for the 16S rRNA gene using the primers and conditions previously described [51]. The strain *E. coli* Nissle 1917 was used as a positive control.

### Pathogenetic Characterization of Isolates

Before evaluating the antibiotic resistance profile of the 25 newly isolated *E. coli* strains, we removed those that possessed some genes typically observed in microorganisms considered potentially pathogenic. By multiplex PCR, we analyzed the following genes: shiga-like toxin (*stx1* and *stx2*) [52]; intimin A (*eaeA*) found in enterohemorrhagic *E. coli* (EHEC) [53]; enterohemorrhagic *E. coli* emolysin (EHEC *hlyA*) [54, 55]; verocytotoxins 1 (*vtx1*) and 2 (*vtx2*), characteristic of verocytotoxin-producing *E. coli* (VTEC) [56]; intimin (*eae*) found in enteropathogenic *E. coli* (EPEC) [57]; heat-stable (*estA*) and heat-labile enterotoxin (*eltA*), characteristic of enterotoxigenic *E. coli* (ETEC) [58, 59]; and invasive plasmid antigen (*ipaH*), characteristic of enteroinvasive *E. coli* (EIEC) and *Shigella* spp. [60, 61]. Genes codifying for *cnf-1* (cytotoxic necrotizing factor 1), *sfa* (S fimbriae), and *pap* (p- associated-pilus) [62, 63] were investigated following the protocol described by Farshad and colleagues [64].

### Antibiotic Susceptibility Profiles

The strains of *E. coli* not harboring pathogenic genes were tested for their sensitivity to antibiotics according to CLSI (Clinical and Laboratory Standards Institute) M07 [65]. Microdilutions were performed in BBL Mueller Hinton II Broth (Merck, Milan, Italy), at pH 7.3 ± 0.1. The minimal inhibitory concentrations (MICs) were determined for 16 antibiotics: ampicillin, penicillin, clindamycin, linezolid (range 0.03 to 16 µg/ml), vancomycin, ciprofloxacin (range 0.25 to 128 µg/mL), neomycin, gentamicin, streptomycin (range 0.5 to 256 µg/ml), kanamycin (range 2 to 1024 µg/ml), erythromycin, quinupristin-dalfopristin (range 0.016 to 8 µg/ml), tetracycline, chloramphenicol, rifampicin, and trimethoprim (range 0.125 to 64 µg/ml). The antibiotic resistance profiles of strains were evaluated based on the European Food Safety Authority (EFSA) guidelines [66]. To determine the susceptibility or resistance of the newly isolated *E. coli* strains, we made a comparison between the MIC values determined for each antibiotic and the breakpoints described by EFSA*.* Furthermore, cefixime susceptibility was considered due to the importance of establishing the probiotics’ sensitivity to a cephalosporin. *E. coli* Nissle 1917 was included as control.

### Whole Genome Sequencing

DNA extraction, library preparation, and in silico genome analysis were performed on the strains according to methods elsewhere described [67–76]. As also previously reported [77], for the strain *E. coli* 5C, a total amount of ~ 3.6 M (2 × 300 bp) reads was generated by Illumina sequencing. After quality filtering and adapter stripping, ~ 3.3 M (~ 92.3%) high quality sequences remained, accounting for ~ 869 M bases. After filtering, ~ 95.7% of remaining reads showed an average Phred quality score of Q30. Filtered reads were then assembled in contigs with SPAdes. Contigs shorter than 500 bp and below 2 × coverage were discarded. Read mapping showed a mean coverage of 182.59 × and a GC content of 58.39%. After inspecting the BLAST4 results (vs. NCBI database), 40 contigs (97.56%) were assigned to *Escherichia* sp. and considered in the follow-up analysis. Genome assembly evaluation resulted in 40 high-quality contigs, with a total size of 4,712,575 bp and a final GC content of ~ 50.79%. The largest contig was 509,219 bp, and the assembly N50 was 335,055. Genome quality assessment displayed a completeness of ~ 99.93% while using 1207 single-copy orthologous genes and no significant evidence of contaminant contigs. As regards the strain 8C, a total amount of ~ 2.1 M (2 × 300 bp) reads was generated by Illumina sequencing. After quality filtering and adapter stripping, ~ 1.9 M (~ 92.8%) high quality sequences remained, accounting for ~ 505 M bases. After filtering, ~ 96% of remaining reads showed an average Phred quality score of Q30. Filtered reads were then assembled in contigs with SPAdes. Contigs shorter than 500 bp and below 2 × coverage were discarded. Read mapping showed a mean coverage of 100 × and a GC content of 50.43%. After inspecting the BLAST results (versus NCBI database), 83 contigs (96.51%) were assigned to *Escherichia* sp. and considered in the follow-up analysis. Genome assembly evaluation resulted in 83 high-quality contigs, with a total size of 4,936,625 bp and a final GC content of ~ 50.57%. The largest contig was 440,212 bp, and the assembly N50 was 147,936. Genome quality assessment displayed a completeness of ~ 99.97% while using 1173 single-copy orthologous genes and no significant evidence of contaminant contigs.

### In Silico pks Detection

The putative presence of genes related to colibactin production was ascertained using a BLAST and mapping approach, respectively. The sequence of target genes was extracted from the annotated genome of *E. coli* Nissle 1917 (NZ_CP058217). For this purpose, a Python script was written to parse the GenBank file of the complete *E. coli* Nissle 1917 genome, and the genes belonging to the *pks* island were found in the following order: *clbA*, *clbR*, *clbB*, *clbC*, *clbD*, *clbE*, *clbF*, *clbG*, *clbH*, *clbI*, *clbJ*, *clbK*, *clbL*, *clbM*, *clbN*, *clbO*, *clbP*, *clbQ*, and *clbS*. Based on the output reference FASTA file, two complementary analyses were conducted, first adopting a BLAST approach and second through read mapping. More in detail, the BLAST search was carried out by building a BLAST database with *pks* island gene sequences, and a local alignment was performed using previously annotated genes from *E. coli* 5C and *E. coli* 8C strains as queries. BLAST tables were then filtered in R using an identity threshold > 80% and coverage > 70% [78]. As a complementary analysis, filtered reads of *E. coli* 5C and 8C strain genomes were mapped against the target genes. More in detail, reads were mapped using BBMap with a minimum read identity of 99% and excluding reads with secondary alignment [79].

### Variant Calling and Comparative Bioinformatic Analysis of *E. coli* 5C Genome and *E. coli* Nissle 1917

Substitutions and insertions/deletions of *E. coli* strain 5C genome versus *E. coli* Nissle 1917 genome as reference were detected using Snippy [80]. Five genome sequences of *E. coli* Nissle strain were downloaded from NCBI (GCF_000333215: 4514, GCF_000714595: 4547, GCF_003546975: 4506, GCF_019967895: 4505, GCF_021559835: 4502). Genomes were sequenced between 2013 and 2022. The FASTA file of assembled genomes included plasmid sequences, which were kept in the analysis since they can harbor AMR elements. Assembled genomic contigs of 5C strain were obtained from GCF_039944155.1 [81].

The presence of putative AMR genes was ascertained using ABRicate [82] against the following databases: NCBI AMRFinderPlus [83], CARD [84], Resfinder [85], ARG-ANNOT [86], MEGARES [87], and EcOH [88]. The databases used for identifying virulence factors were VFDB [89] and *E. coli*_VF [90]. ABRicate was used with default parameters, and only genes with at least 80% identity and 80% coverage were considered as legitimate hits. AMR and VF data were imported in R [91], processed with the dplyr package [92] and plotted using the ggplot2 package [93]. A pangenome analysis of *E. coli* 5C and *E. coli* Nissle 1917 was conducted with PPanGGOLiN [94]. Coding sequences (CDS) were parsed with Prodigal [95], tRNAs were found using ARAGORN [96], and rRNAs were annotated using the Infernal command-line tools coupled with the HMM of the bacterial and archaeal rRNAs downloaded from RFAM [97]. Then, the CDS overlapping any RNA genes were deleted, as they are usually false positive calls [98]. All proteins were clustered using MMseqs2 [99] according to 80% identity and 80% coverage; gene families were assigned to “persistent,” “shell,” or “cloud” partitions and saved.

### Prophages Detection in *E. coli* Strains

The PHASTEST tool (Phage Search Tool for *Escherichia coli*), a freely accessible [100] and user-friendly online tool that can identify and classify prophages and other phage-related sequences in bacterial genomes, was used to detect phage sequences in the *E. coli* genomes [101].

### Rep-PCR Analysis

Rep-PCR is a molecular technique applied to genomic DNA that allows the genetic profile of bacterial strains belonging to the same species to be compared. We performed Rep-PCR reactions using BoxAR1 (5′- CTACGGCAAGGCGACCTGACG-3′) using thermal cycles and PCR conditions following the method described by Estrada et al. [102]. The genomic DNA of both *E. coli* Nissle 1917 and *E. coli* 5C was analyzed by Rep-PCR using the (GTG)5 (5′-GTGGTGGTGGTGGTG-3′) primer and the REP1R-Dt/REP2-Dt couple of primers (REP1R-Dt: 5′-IIINCGNCGNATCNGGC-3′ and REP2-Dt: 5′- NCGNCTTATCNGGCCTAC-3′) [103] in agreement with the thermal protocol previously described [104]. We loaded Rep-PCR amplification products on agarose gel 2.5% (weight/volume).

### Serotyping

To address the *E. coli* 5C serotyping determination, we first run the ECTyper script for the in silico prediction [105, 106]. Default parameters were used to complete the analysis. To validate the result obtained, we also made the serotyping determination at the Statens Serum Institut (Copenhagen, Denmark) according to a method previously described [107].

### Phylotyping

To address the *E. coli* 5C phylotyping determination, we adopt a previously described quadruplex method that exploits aligning the sequences available in Genbank for the *chuA*, *yjaA*, tspE*4.c2*, *arpA*, and *trpA* genes [108].

### Resistance to Digestion Process

The strain of *E. coli* 5C was cultured on BHI (Brain Heart Infusion) broth (Kairosafe, Trieste, Italy). To evaluate its tolerance to simulated gastric juice (SGJ), the broth culture was centrifuged, the supernatant was discarded, and the pellet was resuspended in 10 ml of sterile SGJ (pH = 1.8/3.4 ± 0.1). The ingredients (expressed as mM) contained in the SGJ composition are sodium taurocholate (0.08), phospholipids (0.02), sodium (34), and chlorine (59). The so-prepared tube was incubated at 37 °C; aliquots of 1 ml were removed and serially diluted at three different time points (T0, T30, and T60 min) for determination of total viable counts. The medium chosen for the viable counts was the BHI agar. All plates were incubated for 24 h at 37 °C under aerobic conditions.

Results are expressed as two percentages using the formula *P* = (µ/M) × 100, where *P* is the percentage of resistance of the microbial strain to the simulated gastric juice; *µ* is the count of live *E. coli* cells in the test sample (in log10) after 30 or 60 (respectively T30 and T60) min of incubation at 37 °C with the simulated gastric juice, and nM is the count of *E. coli* (in log10) at the time of preparation of the cell suspension (T0).

We then performed the tolerance test in simulated intestinal juice (SIJ; pH = 8 ± 0.1) after 240 and 360 min of contact. The resistance assessment consists of viable counts performed at different sampling times: at time zero T0 and after 240 (T240) and 360 (T360) min of incubation at 37 °C. The strains under analysis were grown in BHI broth for 24 h at 37 °C under aerobic conditions; then, the cultures were centrifuged, the supernatants discarded, and the bacterial pellets resuspended in 10 ml of SIJ. One ml of the initial suspensions was serially diluted at time zero and at subsequent experimental times T240 and T360. The ingredients (expressed as g/L) contained in the SIJ composition are pancreatin (1), ox-bile (3), and sodium chloride (9). Calculations for intestinal survival are expressed as those for gastric resistance. *E. coli* Nissle strain was used as a reference control. All assays were performed in triplicates.

### Adhesion of *E. coli* to Human Cell Lines

*E. coli* 5C adhesion to human epithelial cell line HT29-MTX derived from the human colon was assessed. Briefly, the HT29-MTX cell line was routinely cultured in High Glucose DMEM (Dulbecco’s modified eagle medium) supplemented with 10% FBS (fetal bovine serum) and antibiotic at 37 °C with 5% CO2 (all sera and media Euroclone, Pero, Milan, Italy). Before the adhesion assay, cells were rinsed with Hank’s balanced salt solution, trypsinized, counted, and seeded in a 24-well plate (2.5 × 10^5^ cell/well). The seeded 24-well plate was incubated for 48 h at 37 °C with 5% CO2 until cells reached the confluence. The day before the test, *E. coli* strains were inoculated in BHI broth and cultured for 24 h at 37 °C under an aerobic atmosphere. On the day of the adhesion test, the wells seeded with cell lines were checked for confluence (85% or more) and washed with Hank’s balanced salt solution. Meanwhile, the strains were prepared for the adhesion assay by washing with sterile distilled water and resuspended in 1% FBS-supplemented DMEM medium. The so-obtained inocula, prepared at a concentration of 1 × 10^6^ CFU/ml, were serially diluted and plated to determine the viable count of microorganisms. Human cells and bacteria were co-incubated for 60 min at 37 °C with 5% CO2, with a multiplicity of infection (MOI) between microorganisms and human cells in the ratio 5:1. Following incubation, the medium was removed from the infected eukaryotic cells, and the monolayer was washed to discard unbonded bacteria. The monolayer was trypsinized, and the suspension of *E. coli* and human cell debris was serially diluted, plated on BHI agar, and incubated for 24 h at 37 °C under aerobic conditions. Percentages of adhesion were calculated considering viable raw count data with the formula *P* = (*µ*/*M*) × 100, where *P* represents the adhesion percentage of *E. coli* to the HT29-MTX cell line, *µ* represents the viable count of analyzed strains bonded to the human HT29-MTX cell line expressed as a logarithmic value, and *M* represents the viable count of analyzed strains transformed as a logarithmic value of the inoculum. *E. coli* strain Nissle 1917 was used as a reference control. Tests were assayed in triplicates.

### Histamine Release

In order to promote the enzyme induction before the screening test, *E. coli* 5C and *E. coli* Nissle 1917 were subcultured 5 times in Nutrient Standard (Millipore 1,07882; Merck, Darmstadt, Germany) containing 0.1% of histidine monohydrochloride as a precursor amino acid, supplemented with 0.005% of pyridoxal-5-phosphate [109]. After 5 days, overnight cultures were centrifuged at 2500 rpm for 15 min, and supernatants were collected. Then, supernatants were analyzed through HPLC. Supernatants were dosed for histamine content three times. Positive histamine-spiked control and blank were included in the analysis.

### Assessment of Acetate Production

Acetic acid production was determined for *E. coli* 5C and *E. coli* Nissle 1917 by a colorimetric commercial kit supplied by Megazyme (Michigan, USA) following the manufacturer’s instruction. The acetate release in the supernatants was quantified after overnight incubation at 37 °C in Nutrient Broth (Liofilchem, Teramo, Italy) under aerobic and anaerobic atmosphere. The positive control included in the kit and the blank sample were run alongside the samples.

### *E. coli* Antipathogenic Activity

*E. coli* strains (5C and Nissle 1917), *E. faecium* (ATCC 19434), *E. faecalis* (ATCC 19433), *E. cloacae* (ATCC 13047), *K. pneumoniae* (ATCC 25955), *K. aerogenes* (ATCC 13048), *C. sakazakii* (ATCC 29544), *S. enterica* (serovar Abony NCTC 6017), and *S. enterica* (serovar typhimurium DSM 5569) were grown in LAPTg (Lactose, Tryptone, Peptone, Tween 80, glucose) broth and incubated aerobically for 24 h at 37 °C. The activity of *E. coli* strains against pathogens was assessed as previously reported [26] by means of coculture in LAPTg medium. Fifty microliter of an overnight culture of the putative probiotic *E. coli* strain and one pathogen culture were incubated in LAPTg medium for an overnight period. One milliliter of these suspensions was serially diluted and plated on Brilliance™ UTI Clarity (Oxoid, Basingstoke, UK), except for *S. enterica* strains that were plated on MacConkey agar (Oxoid, Basingstoke, UK) and incubated for 24 h at 37 °C under aerobic conditions. Pathogens were also incubated without the presence of the putative probiotic strain as a reference undisturbed control. An ANOVA followed by Dunnett’s T3 multiple comparison test versus the pathogen alone was performed (*p* < 0.05).

### Assessment of the Immunomodulatory Properties *E. coli* Strains

Human peripheral blood mononuclear cells (hPBMCs; Lonza, Basel, Swisse) were stimulated with viable cells of each strain (*E. coli* 5C and *E. coli* Nissle 1917) for 24 h at an MOI ratio of 1:10 (hPBMCs: bacteria). Upon arrival, following the manufacturer’s instructions, the cells were immediately thawed following the specific protocol and were plated in a 24-multiwell-plate at the concentration of 1 × 10^6^ cell/ml in RPMI (Roswell Park Memorial Institute) medium added with 10% heat-inactivated FBS, 2 mM L-glutamine, and 50 µg/ml gentamicin. Cells were left undisturbed for 24 h; then, we started the experiment. We evaluated the immunomodulation potential of the strains by stimulating hPBMCs with probiotic viable cells for 24 h in basal and inflamed (LPS-triggered) conditions (0.1 µg/ml of LPS). At the end of this period, supernatants were collected and used for cytokine quantification. IL-12p70 was quantified to evaluate the pro-inflammatory potential of the strains, whereas IL-10 was used as an anti-inflammatory marker. Each condition was tested in duplicate to obtain a biological replicate. For both IL-12p70 and IL-10 quantification induced by the novel isolated strains, an ANOVA followed by Dunnett’s T3 multiple comparison test versus unstimulated cells was performed (*p* < 0.05 versus negative control). A *t*-test between the two strains (*E. coli* 5C versus *E. coli* Nissle 1917) was also performed.

### Pks Gene Cluster Detection in Finished Products Through PCR

To confirm the absence of the *pks* gene in *E. coli* A0 34/86 and to evaluate its possible presence in DSM 17252, we have respectively analyzed the bacterial cells contained in Colinfant® New Born and the mixture of *E. coli* strains (G1/2, G3/10, G4/9, G5, G6/7, G8) contained in Symbioflor® 2 through PCR, according to the method described previously [110]. We have used as analytical references the strains *E. coli* Nissle 1917 (positive control) and *E. coli* 5C (negative control). The *pks* gene cluster was searched using primers targeting four genes (*clbA*, *clbB*, *clbN*, and *clbQ*) [107]. After incubating overnight, the strains Nissle 1917 and 5C were resuspended in 20 µl of Microlysis Plus (Microzone, Stourbridge, UK) and lysed according to the manufacturer’s instructions. One ml of Colinfant® New Born and 1 ml of Symbioflor® 2 were centrifuged, the supernatants decanted, and the pellets lysed through the Microlysis Plus protocol. The PCR amplification was performed as previously described [111].

## Results

### Species-Specific Identification, Pathogenetic Characterization and Antibiotic Sensitivity Profiles

Stool samples were collected from infant healthy volunteers, and after the isolation process, 25 strains of presumptive *E. coli* were recovered. All of them were confirmed to belong to *E. coli* species by species-specific PCR [50]. All isolates were tested for the presence of 13 potential virulence factor genes, as described in the “Materials and Methods” section. As shown in Table 1, only two strains (namely 5C and 8C) out of the 25 initial isolates were positively selected, being completely devoid of these virulence factor genes, which were, on the opposite, retrieved in the other 23 strains. As shown in Table 2, both these two strains, as well as the strain Nissle 1917, were found to be safe according to the EFSA probiotics guidelines concerning their antibiotic-susceptibility profile. Also, versus cefixime, a representative of the cephalosporin class not included in the EFSA panel, the strains resulted to be susceptible according to a set cut-off recently demonstrated [112].

**Table 1:**
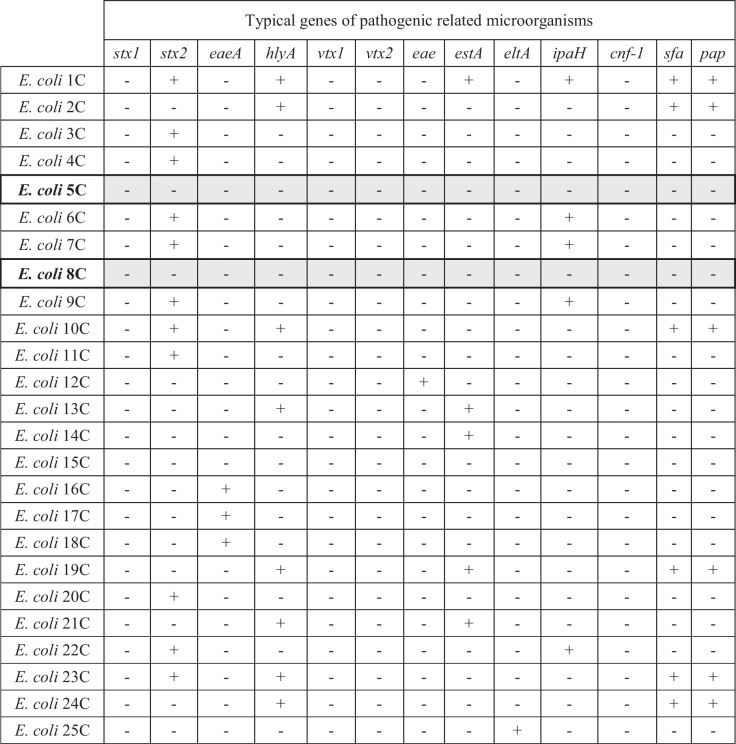
Potential pathogenic related genes screening

+ : positive amplification fragment; -: negative amplification band. *stx1* and *stx2*: shiga toxin, bacterial toxins inhibiting cellular protein synthesis; *eaeA*: intimin, a toxin causing attaching-effacing lesions in the gut mucosa found in enterohemorrhagic *E. coli* (EHEC); *hlyA*: an emolysin found in EHEC *E. coli*; *vtx1* and *vtx2*: verocytotoxins found in verocytotoxin-producing *E. coli* (VTEC); *eae*: intimin found in enteropathogenic *E. coli* (EPEC); *estA* and *eltA*: heat-stable and heat-labile enterotoxins, characteristic of enterotoxigenic *E. coli* (ETEC); *ipaH*: invasive plasmid antigen, characteristic of enteroinvasive *E. coli* (EIEC) and *Shigella* spp.; *cnf-1*: cytotoxic necrotizing factor-1 found in uropathogenic *E. coli* (UPEC); *sfa*: *S-fimbriae* found in UPEC *E. coli*; *pap*: p-associated-pilus, a virulence factor associated with pyelonephritis (for appropriate references, see “Materials and Methods” section)

**Table 2:** Antibiotic resistance profiles of strains 5C and 8C, in comparison with *E. coli* Nissle 1917

MIC value (µg/ml)				
	*E. coli* Nissle	*E. coli* 5C	*E. coli* 8C	EFSA cut-off
Gentamicin	1	2	1	2
Kanamycin	8	4	8	8
Streptomycin	16	16	8	16
Neomycin	2	2	2	nr
Tetracycline	4	8	8	8
Erythromycin	> 8	> 8	> 8	nr
Clindamycin	> 16	> 16	> 16	nr
Chloramphenicol	8	8	16	nr
Ampicillin	4	4	4	8
Penicillin	> 16	> 16	> 16	nr
Vancomycin	> 128	> 128	> 128	nr
Quinupristin-dalfopristin	> 8	> 8	> 8	nr
Linezolid	> 16	> 16	> 16	nr
Trimethoprim	1	16	2	nr
Ciprofloxacin	< 0.25	< 0.25	< 0.25	0,06
Rifampicin	16	32	32	nr
	*E. coli* Nissle	*E. coli* 5C	*E. coli* 8C	Set cut-off*
Cefixime	0.25	0.25	0.5	1

*nr* not requested

*According to references no. 108

### Genome Analysis, Comparative Genomics, and REP-PCR

According to the Achtman MLST (multi-locus sequence typing) scheme, analysis demonstrated that all (strains 5C, 8C, and Nissle 1917) genomes belonged to the *E. coli* species, with ST73 [40] being the most prevalent sequence types (STs) identified in Nissle 1917 and not present in strains 5C and 8C. Functional genetic annotation revealed that neither antibiotic resistance genes nor toxin-related genes were detected in prophage sequences observed in the three strains. A detailed review of the MLST analysis performed on the genomes of the three strains, including putative virulence factors, efflux pumps, and plasmids is shown in Online Resource 1. To better identify and classify prophages and other phage-related sequences in bacterial genomes, we have used the PHASTEST tool [100, 101]. As regards *E. coli* 5C, we have found three complete sequences: PHAGE_Escher_HK639 (NCBI Accession: NC_016158), PHAGE_Entero_HK629 (NCBI Accession: NC_019711), and PHAGE_Entero_fiAA91 (NCBI Accession: NC_022750). As regards *E. coli* 8C, we found one intact and two questionable, since incomplete, sequences. They are respectively phage_Entero_DE3 (NCBI Accession: NC_042057), phage_Salmon_118970_sal3 (NCBI Accession: NC_031940), and phage_Entero_lambda (NCBI Accession: NC_001416). As regards *E. coli* Nissle 1917, the analysis performed on GCF000333215 demonstrated a too short sequence to be analyzed by the PHASTEST tool. The analysis on GCF000714595 demonstrated two intact prophage sequences: phage_Entero_lambda (NCBI Accession: NC_001416), also found in strain 8C, and phage_Entero_c_1 (NCBI Accession: NC_019706). Both correspond to bacteriophages harmless for humans, specifically infecting only *E. coli* strains. The analysis on GCF003546975 demonstrated two intact and one incomplete sequences, respectively, phage_Entero_DE3 (NCBI Accession: NC_042057), also found in strain 8C; phage_Entero_c_1 (NCBI Accession: NC_019706), also shown by GCF000714595; and Phage_Gifsy_1 (NCBI Accession: NC_010392), a lambda-like phage affecting the *Salmonella* genus. The analysis performed on GCF019967895 found two complete sequences: phage_Entero_lambda (NCBI Accession: NC_001416) and phage_Entero_c_1 (NCBI Accession: NC_019706). Both the analysis performed on GCF021559835 and GCF043228065 demonstrated two complete and one incomplete sequences: phage_Entero_lambda (NCBI Accession: NC_001416), phage_Entero_c_1 (NCBI Accession: NC_019706), and phage_Gifsy_1 (NCBI Accession: NC_010392). A detailed review performed on the PHASTEST tool is shown in Online Resource 2.

**Fig. 2:**
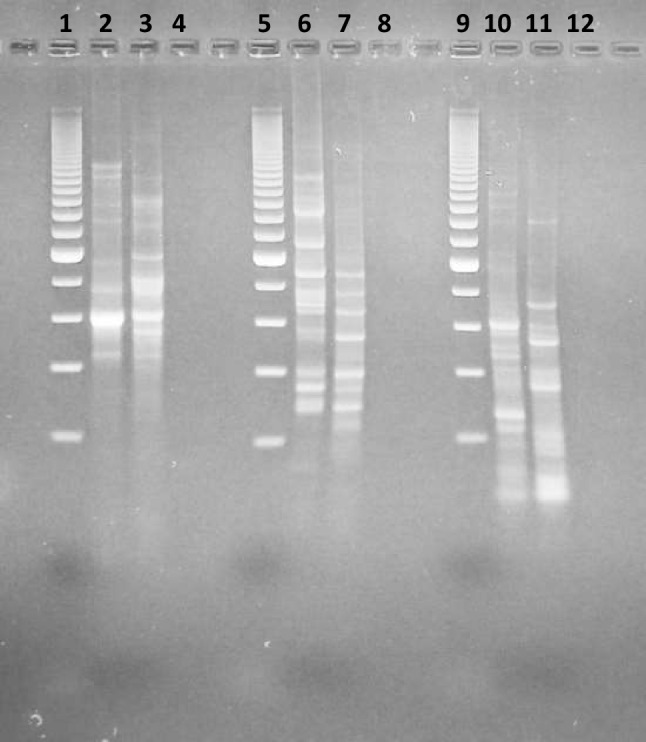
*E. coli* strains genomic fingerprinting. 1,5,9: Marker 200 pb; 2: E. coli Nissle 1917 with BoxAR1 primer; 3: *E. coli* 5C with BoxAR1 primer; 6: *E. coli* Nissle 1917 with GTG5 primer; 7: *E. coli* 5C with GTG5 primer; 10: *E. coli* Nissle 1917 with Rep1,2 Dt primer; 11: *E. coli* 5C with Rep1,2 Dt primer; 4,8,12: Negative Template Control

Main results of the genome analysis of the two strains of *E. coli* 5C and 8C are summarized in Table 3. As also previously reported [77], for the strain *E. coli* 5C, gene prediction and annotation identified 4664 genes, among which 4357 were coding sequences. Moreover, no genes belonging to the *pks* island for colibactin production nor plasmids were identified [77]. As regards the strain *E. coli* 8C, gene prediction and annotation identified 4998 genes, among which 4602 were coding sequences. As for strain 5C, also for strain 8C, no genes belonging to the *pks* island for colibactin production were identified. In strain 8C, two contigs were identified as plasmid sequences. The presence of plasmids was also confirmed by BLAST search [113]. Plasmid contigs were found to host putative virulence factors (Online Resource 3). Due to this feature, the *E. coli* 8C isolate was discarded. Differently, the whole draft sequence of *E. coli* strain 5C was deposited in Genbank with the BioProject no. PRJNA1114436, and the strain was safely deposited at the LMG-BCCM under the LMG S-33222 number [77]. The entire genome shotgun project of *E. coli* 5C has been submitted to NCBI with the BioProject accession number PRJNA1114436 and the corresponding GenBank accession number JBDPNK000000000.1. The raw reads are available in the Sequence Read Archive under accession number SRR29289048, and the assembly can be accessed using the accession number GCA_039944155.1. Phylogenetic placement analysis performed on genes *fumC* and *fimH*, both in strain 5C and in strain Nissle 1917, used as control (Online Resource 4), confirmed that the strain *E. coli* 5C belongs to the *E. coli* species [114]. In detail, the strain 5C harbors the alleles *fumC95* and *fimH121*, and the strain Nissle 1917, *fumC24* and *fimH30*. Noteworthy, this last allele found in strain Nissle 1917 is described as a possible pathogenic feature found in antibiotic-resistant *E. coli* [115]. The in silico evaluation of substitutions and insertions/deletions in the genome of *E. coli* strain 5C, versus the genome of *E. coli* Nissle 1917 used as reference, demonstrated a total amount of 82,332 variants detected. In detail, comparative analysis between the strain 5C and the strain Nissle 1917 shows substantial similarity in terms of antimicrobial resistance genes, where most of these putative resistances are species-specific elements attributable to the *E. coli* non-susceptibility to antibiotics commonly effective against Gram-positive bacteria (Online Resource 5) [26]. Similarly, the number of putative virulence factors, to be considered elements of potential bacterial fitness, is superimposable between the strain 5C and the strain Nissle 1917. Indeed, both strains harbor genes related to adhesins, iron uptake, and flagellar biosynthesis and motility, which are important for bacterial survival and spread, but that are not necessarily related to virulence and/or pathogenicity (Online Resource 6). As shown in the Venn diagram superimposing the 5C and the complete Nissle 1917 genomes (Online Resource 7), the number of coding sequences (CDS) common to both strains’ genomes was 3555. The number of CDS unique to the 5C strain was 744. Genes specific to strain *E. coli* 5C were further analyzed, and functional annotation was performed as described [97]. The number of corresponding proteins that found a match in the eggNOG database was 687. The Clusters of Orthologous Groups (COG) categories summarizing the proteins unique to strain *E. coli* 5C covered a wide variety of metabolic functions, as shown in Online Resource 8. Many fitness factors seem to be apparently specific for the *E. coli* strain 5C (Online Resource 9), which demonstrates a certain uniqueness (Online Resource 10). A list of genetic features characterizing the strain *E. coli* 5C, in comparison with those already known and reported for the strain *E. coli* Nissle 1917 [26], is reported in Table 4. As regards to adhesion molecules (types of fimbria) [26] *E. coli* 5C is positive forthe *fim* (common type I fimbria; F1A) and *csgA* (Curli fimbria) genes, but negative for the *foc* (F1C fimbria), *sfa* (S-type fimbria), *pap* (P-type fimbria), and *cfa* I/II (colonizing factor antigens I and II) genes (absence of *sfa* and *pap* genes is also reported in Table 1). As regards the gene *rfaH*, a transcriptional regulation factor enhancing the colonizing capability of *E. coli* strains and their capability to resist bile salts [26, 33], the strain *E. coli* 5C was found to have it. Regarding the genes of the most important toxins possibly found in *E. coli* [26], like α-Hemolysin (*hly*), CNF I (cytotoxic necrotizing factor I; *cnf* I), H-LT (heat-labile enterotoxin; *etx*), H-ST (heat-stable enterotoxin; *est*), and shiga toxins (*stx*), some of them also reported in Table 1, they were all not found in strain *E. coli* 5C. As regards iron acquisition (siderophores) [26], we have evaluated the following genes: *ent* (Enterobactin), *iro* (Salmochelin), *iuc*/*aer* (Aerobactin), *ybt* (Yersiniabactin), and *chu* (Hemin uptake system). They were all absent in *E. coli* 5C. Finally, as regards the citrate-dependent iron acquisition capability, both the *strain E. coli* 5C and Nissle 1917 demonstrated the same positive superimposable gene pattern (Online Resource 11). As regards to bacteriocin genes, the strain Nissle 1917, besides the already described two microcins, known as M and H47 [31, 32], demonstrates the presence of one colicin and one bottromycin [116], both not phenotypically expressed. The last gene, bottromycin, is genetically present, but likely not phenotypically expressed, also in the strain 5C. It is possible that the bottromycin gene is interrupted in both strains (Online Resource 12). Lastly, as shown in Fig. 2, the *E. coli* 5C genomic DNA was amplified through REP-PCR, and the *E. coli* Nissle 1917 was used as control. The molecular fingerprinting of the two strains was different for all the REP-PCR reactions, indicating their different genomic patterns.

**Table 3:** Main features derived from the genomic analysis of *E. coli* strains 5C and 8C

	Total size* (bp)	GC content* (%)	Genes	Coding sequences	*pks*	Plasmids number	Virulence factors on plasmids
*E. coli* 5C	4,712,575	50.79	4664	4357	Negative	0	/
*E. coli* 8C	4,936,625	50.57	4998	4602	Negative	2	Positive

*Data reported in the “Materials and Methods” section

**Table 4.**
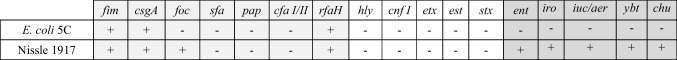
Genetic features concerning adhesion and colonization of the gut and bladder (light grey), toxins release (white) and iron acquisition properties (grey) derived from the genomic analysis of *E. coli* strains 5C (in comparison with *E. coli* Nissle 1917)

*fim*: common type 1 fimbria; *csgA*: Curli fimbria; *foc*: F1C fimbria; *sfa*: S-type fimbria; *pap*: P-type fimbria; *cfa* I/II (colonization factor antigens I/II); *rfaH*: a transcriptional antiterminator gene; *hly*: emolysin; *cnf-1*: cytotoxic necrotizing factor-1; *etx*: heat-labile enterotoxin; *est*: heat-stable enterotoxin; *stx*: shiga toxin; *ent*: enterobactin; *iro*: salmochelin; *iuc/aer*: aerobactin; *ybt*: yersiniabactin; *chu*: hemin uptake system. Data concerning the strain *E. coli* Nissle 1917 are both described in ref. [26] and further confirmed by us by gene alignment

### Serotyping and Phylotyping

The serotyping determination of strain *E. coli* 5C, performed with the ECTyper script for the in silico prediction, further validated by the SerotypeFinder for whole-genome sequencing-based O and H typing, gold standard for *E. coli* serotyping [117], confirmed the presence of the O173 antigen (LPS) and the H1 antigen (flagella). The K antigen (capsule) does not correspond to any of the known ones. The *E. coli* 5C has therefore the following serovar: O173:KNT:H1 (NT-no typable, Online Resource 13). The serovar for *E. coli* Nissle 1917 is O6:K5:H1 [26]. As regards phylotyping, among the five genes considered in the quadruplex method [108], *E. coli* 5C matches only with the *ArpA* gene, therefore belonging to the phylogroup A (Online Resource 14).

### Simulated Gastric and Intestinal Resistances, Histamine release, and Acetic Acid Production

Some probiotic properties of the *E. coli* strain 5C, like the gastric and intestinal resistances, the histamine release, and the acetic acid production, are reported in Table 5. The strain 5C and the strain Nissle 1917 similarly survived both in the presence of simulated gastric juice (SGJ) and intestinal juices (SIJ) at pH 3.4, but strain 5C displayed higher tolerance at a lower pH, like 1.8. The adhesive properties of the strain 5C seemed to be more pronounced if compared to the control strain (Nissle 1917). Furthermore, the release of histamine was slightly increased for the strain 5C strain even if at a very low level and not significantly different from Nissle 1917. The acetate production is comparable between the two strains and seemed not to be affected by the presence of oxygen. The resistance of the newly isolated *E. coli* 5C to the digestion process is comparable to that of the strain Nissle 1917.

**Table 5:** Simulated gastric and intestinal resistances, histamine and acetate release for *E. coli* Nissle 1917 and *E. coli* 5C

	SGJ (% ± SD) pH 3.4	SGJ (% ± SD) pH 1.8	SIJ (% ± SD) pH 8	Adhesion (% ± SD)	Histamine (ppm ± SD)	Acetic acid (g/L ± SD) aerobic atmosphere	Acetic acid (g/L ± SD) anaerobic atmosphere
*E. coli* Nissle 1917	96.3 ± 4.5	35.3 ± 8.2	97 ± 2.8	58 ± 5	4 ± 2.2	0.362 ± 0.05	0.373 ± 0.04
*E. coli* 5C	99.7 ± 3.7	67.9 ± 7.4	98.3 ± 1.1	84 ± 4	19 ± 3.6	0.366 ± 0.06	0.384 ± 0.03

*SGJ* simulated gastric juice,; *SIJ* simulated intestinal juice, *ppm* parts per million

### *E. coli* 5C Antipathogenic Activity

**Fig. 3:**
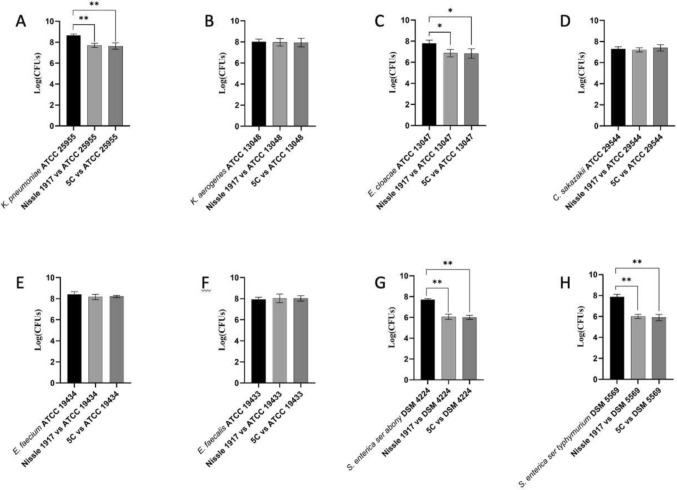
Antipathogenic activity exerted by the strain *E. coli* 5C compared to *E. coli* Nissle 1917 against selected pathogens. Black bars represented the undisturbed pathogen growth, while light and dark gray bars displayed the effect of the co-culture of probiotic and pathogen strains. One-way ANOVA with multiple comparisons test versus control: *p < 0.05 and **p < 0.01

To evaluate the anti-pathogens’ potential role exerted by the strain *E. coli* 5C, we have tested it against strains representative of *K. pneumoniae* (ATCC 25955), *K. aerogenes* (ATCC 13048), *E. cloacae* (ATCC 13047), *C. sakazakii* (ATCC 29544), *E. faecium* (ATCC 19434), *E. faecalis* (ATCC 19433), *S. enterica* (serovar Abony NCTC 6017), and *S. enterica* (serovar typhimurium DSM 5569). As a reference, we have used the strain *E. coli* Nissle 1917. According to the results shown in Fig. 3, the strain *E. coli* 5C displayed a significant antipathogenic activity against *K. pneumoniae* (Fig. 3A), *E. cloacae* (Fig. 3C), and the two serovars of *S. enterica* (Fig. 3G, H). The same performances were registered for the reference *E. coli* Nissle 1917.

### *E. coli* Strain Immunomodulation Properties

**Fig. 4:**
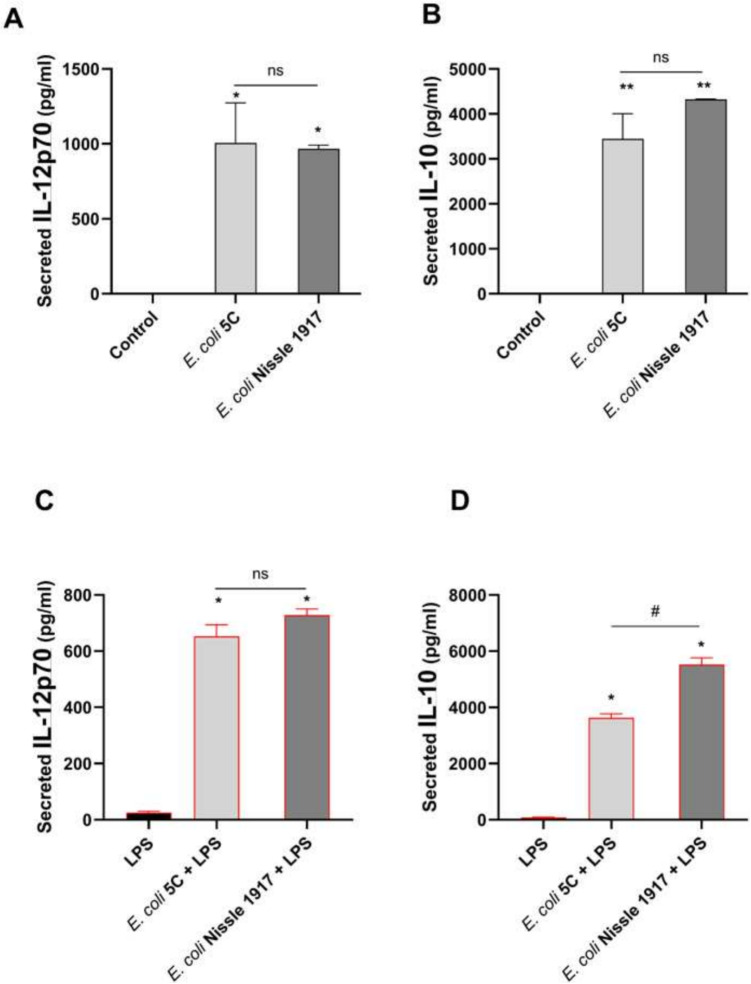
Immune modulatory responses in basal (**A**, **B**) and in LPS-triggered (**C**, **D**) conditions exerted by *E. coli* 5C (versus *E. coli* Nissle 1917). **A**, **B**: One-way ANOVA with multiple comparisons test versus control: *p < 0.05 and **p < 0.01. *t*-test between the two strains does not reach statistical significance. **C**, **D**: One-way ANOVA with multiple comparisons test versus LPS-stimulated cells: *p < 0.05 and **p < 0.01. *t*-test between 5C and Nissle strains: # < 0.05

To evaluate the potential ability of the strain *E. coli* 5C in modulating the immune response, we stimulated hPBMCs with this strain for 24 h and quantified the secreted cytokines IL-12p70 and IL-10 by ELISA technique. *E. coli* Nissle 1917 was used as a control strain. In basal conditions, the two strains exerted similar immune modulatory roles (Fig. 4A, B), inducing the release of significant levels of IL-12p70 and IL-10 cytokines but without significant differences. In LPS-triggered conditions (Fig. 4C), the strain *E. coli* 5C induced the release of IL-12p70 in a comparable manner to that induced by the control strain. As shown in Fig. 4D, the strain *E. coli* 5C was able to induce the release of the anti-inflammatory IL-10 cytokine even in these LPS-triggered conditions, but the effect seems to be lower than the one yielded by the control strain.

### Pks Gene Cluster Detection in DSM 17252

We have then analyzed the mixture of *E. coli* strains (G1/2, G3/10, G4/9, G5, G6/7, and G8) contained in Symbioflor® 2 and the probiotic contained in Colinfant® New Born through PCR to highlight the possible presence of the *pks* gene. The analysis has revealed that the *E. coli* mixture is positive for colibactin (Online Resource 15). The low intensity of the signal observed in the electrophoretic run is due to the low bacterial load, relative to the live and viable bacteria formulated in the mixture and found in the finished product. The manufacturer declares, in fact, to inactivate some of the strains in the mixture, making them non-viable. As expected, strain *E. coli* Nissle 1917 also resulted to be positive, while *E. coli* A0 34/86 and *E. coli* 5C resulted to be negative for *pks* detection.

## Discussion

Although the first *E. coli* ever isolated (by T. Escherich in 1885, today known as strain NCTC86) was a non-pathogenic strain [118], and although the species is abundantly present in most newborns and around 1% in 90% of healthy adults [119, 120], most of what we know about this species comes from studies performed on pathogenic strains, and little is known about which biotic and abiotic factors influence the presence and distribution of non-pathogenic strains in humans. Most non-pathogenic *E. coli* present in the human intestine belong to phylogroups A, B2, and F [121]. Despite understandable emerging issues in probiotic safety, among probiotics used for both generally healthy consumers and in clinical settings, a few belong to the *E. coli* species [122].

### The Issue of Colibactin-Releasing *E. coli* Strains

The most investigated *E. coli* probiotic is the strain Nissle 1917 [123]. This strain has been used for over a century with few reports of adverse events reported in the literature [124]. However, like other members of the B2 phylogenetic group, Nissle 1917 contains the *pks* gene cluster that encodes a series of enzymes that produce a DNA alkylating and double-strand break-inducing genotoxin known as colibactin [23]. Concern arises because, in several mouse models, other colibactin-producing *E. coli* have been observed to promote the development of colorectal cancer [125–131], and human organoids exposed to certain *pks*-positive *E. coli* exhibit colibactin-dependent mutational signatures also found in human colorectal cancers [35]. Nissle 1917 was initially thought not to be genotoxic [131]. However, colibactin was then detected in Nissle 1917 supernatants, and cell lines exposed to such supernatants were found to have clear DNA mutations [132]. In germ-free mice administered with Nissle 1917, intestinal epithelial cells showed evidence of DNA damage [132], which was not observed when mice were inoculated with variants of Nissle 1917 carrying mutations in *clbA* or *clbP*, genes encoded within the *pks* cluster and which are directly involved in colibactin synthesis [47, 133]. To the best of our knowledge, there are no data linking Nissle 1917 with colorectal cancer in humans. Since the risk still exists, a “solution” suggested by many authors is the removal of the *pks* island [134]. To date, such a modified strain of Nissle 1917 for human use has not yet been produced.

### Selection of a Probiotic *E. coli* Strains Not-Releasing Colibactin

Another chance is to select a new strain of *E. coli*, possibly with similar probiotic features and devoid of the *pks* cluster. With this aim in 2020, we have started selecting from infant stool a new strain of *E. coli*. We decided to start from newborn stool (no older than 6 months of life) because, in that fecal context, it is easier to find a significant number of *E. coli* strains. In healthy newborns, *E. coli* can account, on average, for 15% of the entire gut microbiota and up to 40% in some subjects [135]. We then decided to start from those newborns that had not undergone antibiotic therapy to avoid finding potentially antibiotic-resistant bacteria. Of course, newborn feces do not guarantee finding *pks*-negative *E. coli* strains. Scientific literature has shown that between 27 and 33% of *E. coli* detectable in the feces of European newborns are pks-positive [136–138]. However, in consideration of the greater possibility of finding *E. coli* strains in newborns compared to adults [119, 120], we decided to opt for newborns as a possible source of *E. coli*. As shown in the “Results” section, from an initial list of 25 potential candidates, on the basis of the results obtained by screening them for a wide pattern of virulence factors, we have first selected two strains, *E. coli* 5C and 8C (both *pks*-negative and antibiotic-sensitive), preferring then the strain 5C one as the strain 8C was found to contain a plasmid carrying potential virulence factors.

### Detection of Prophage Regions by the PHASTEST Tool

Prophages analysis in *E. coli* strains should be considered an essential aspect when characterizing a potential probiotic. In strain *E. coli* 5C, we have found only three complete sequences corresponding to phage_Escher_HK639, phage_Entero_HK629, and phage_Entero_fiAA91. The first, the phage HK639, is a double-stranded DNA virus that infects *E. coli*. It is classified as a member of the Caudoviricetes class, specifically within the unclassified Caudoviricetes order. It does not encode any known toxins or virulence factors that could harm humans [139]. The second, the phage HK629, is also considered not dangerous for humans or animals. It is not inherently present in all *E. coli* strains, and it can integrate into the bacterial genome under certain conditions [140]. The third, the phage fiAA91, is not dangerous for humans or animals as well, showing similar features as HK629 [141]. According to a recent depiction of human gut-derived prophages, useful in providing a substantial collection of reference sequences for forthcoming human gut phageome-related investigations and potentially enabling better risk assessment of prophage dissemination [142], *E. coli* is one of the bacterial species with the higher prevalence of prophages. Indeed, 97.0% of *E. coli* strains harbor complete prophages, and 87.0% of *E. coli* strains contain prophage regions. This suggests that *E. coli* is particularly prone to prophage integration, which aligns with the observation of multiple prophages in a single *E. coli* genome. For instance, *E. coli* strain M0110 was found to harbor as many as 18 complete prophages. This indicates that *E. coli* genomes can accommodate multiple prophages, which is consistent with the presence of the three phages in the genome of the strain *E. coli* 5C. Nevertheless, some prophages could have the ability to integrate across different bacterial genera, although this is relatively rare (~ 4% of prophages) [142]. However, the presence of multiple prophages in a single *E. coli* genome suggests that these phages may have a narrow host range, specifically targeting *E. coli*. This is also supported by the evidence that the considered phages, found in strain 5C, are all associated with the family Enterobacteriaceae to which *E. coli* belongs. Moreover, the prophages could potentially contribute to the bacterium’s adaptability, pathogenicity, or resistance to environmental stresses. This is particularly relevant given that *E. coli* is a common gut bacterium endowed with many fitness factors. Lastly, the nature of prophages is dynamic. They can switch between lysogenic (integrated) and lytic (active) states. The presence of multiple prophages in a single *E. coli* genome could indicate that these phages are in a lysogenic state, being integrated into the bacterial chromosome and replicating along with the host. However, under certain conditions (e.g., stress), these prophages could be induced into the lytic cycle, leading to the production of phage particles, potentially causing lysis of the bacterial cell.

### Comparison Between the Main Features of *E. coli* Strains 5C and Nissle 1917

The direct genetic comparison between the *E. coli* strains, 5C and Nissle 1917, highlights that they are quite similar (according to the reference databases, the two strains are completely overlapping by 66.9%; Online Resource 4) but with some differences since, always according to the available databases, the strain *E. coli* 5C shows genomic uniqueness by 14.0% (Online Resource 4). They belong to different phylotypes, A-type for strain 5C and B2-type for Nissle 1917. Recent findings have shown that phylotypes A and B2 are the most distant from each other phylogenetically among *E. coli *sensu stricto [107]; that strains responsible for extra-intestinal infection were far more likely to be members of phylogroups B2 than A [143, 144]; and that the B2 phylotype is likely the colibactin-positive most virulent phylogroup among *E. coli* [145]. As regards serovar, *E. coli* Nissle 1917 is characterized by an LPS-type, O6, commonly found in extra-intestinal pathogenic *E. coli* [29, 30, 146]. Moreover, only *E. coli* Nissle 1917 is endowed with F1C fimbria described to allow bacteria to adhere to the urinary tract [147]. Indeed, when transurethrally inoculated into the bladder of female rats, the Nissle 1917 strain determines the presence of colonies both in the bladder and in the renal tissue [26]. In terms of potential probiotic activity, they show superimposable features. Both are antibiotic-sensible, at least according to the antibiotic panel established by EFSA authorities; both similarly resist in simulated gastric and intestinal juice, and both have a similar capability of histamine and acetate release. Both share a similar pattern of anti-pathogenic activity and immunological properties. The only minimal differences observed between the two strains concern a slightly better gastrointestinal resistance of *E. coli* 5C and a slightly better IL-10 response generated in human cells by the presence of *E. coli* 1917. Noteworthy is the difference between the two strains with regard to the presence of the *pks* genetic island.

### *E. coli* 5C, a Putative Safe Probiotic

As shown in our study, the *pks* genetic island is absent in the *E. coli* 5C but present and productive in the *E. coli* Nissle 1917 [39]. Considering that the *E. coli*-based probiotic mixture Symbioflor® 2 is *pks*-positive and Colinfant® New Born is hly- and *cnf-1*-positive, the strain *E. coli* 5C could be considered one of the most, if not the most, safe *E. coli*-based probiotics available for human use. This could also have a significant impact in the oncology field. Colorectal cancer (CRC) is the third most common cancer worldwide. Approximately 2 million people are diagnosed with CRC each year, of which 50% succumb to the disease [148]. Studies on the gut microbiota have recently provided strong evidence of the involvement of intestinal oncobacteria such as *F. nucleatum*, enterotoxigenic *B. fragilis*, and pathogenic *E. coli* strains [149–152]. The negative role of the latter is due (also) to their ability to produce colibactin through the *pks* gene. These *pks*-positive *E. coli* strains have been detected in approximately 20% of healthy individuals, 40% of patients with inflammatory bowel disease, and 60% of patients with CRC and are therefore associated with the inflamed and neoplastic mucosa, where they can promote both the onset and progression of CRC [126, 153, 154]. Noteworthy, human intestinal organoids injected with *pks*-positive *E. coli*, including the strain *E. coli* Nissle 1917, revealed that colibactin promotes a distinct mutational signature that is also detected in around 12% of patients with CRC [39], particularly in patients diagnosed at a young age [35]. Very recent studies indicate that, in contrast to extremely pathogenic strains like *E. coli* 11G5, *E. coli* Nissle 1917 is less oncogenic but may not be considered harmless [155]. This difference in oncogenicity can be explained by observing the actual lower adhesion capabilities to the intestinal epithelium shown by the *E. coli* Nissle 1917 strain when compared to that of the more oncogenic strains [155]. The idea that a probiotic has the genetic capability to produce colibactin is worrisome, and the safe use of *E. coli* Nissle 1917 as a probiotic remains very uncertain, also considering the potential for strain variation due to evolutionary changes and/or horizontal gene transfer and recombination events that could increase its adhesion capability. In our opinion, *pks*-negative strains should therefore always be preferred when considering *E. coli*-based probiotics. Recently, this approach has been preferred by some physicians who have safely and successfully treated patients recurrently suffering from intestinal discomfort following colonoscopy with the strain *E. coli* 5C [156]. With this same application, and with the brand ECP24®, a clinical trial on thousands of patients is being planned and will probably start in 2025.

## Conclusion

Starting from 25 possible candidates, our study allowed us to select an *E. coli* strain (5C; deposited as LMG S-33222) with fitness and probiotic characteristics similar to those of the *E. coli* Nissle 1917, but with a main substantial difference: the absence of the *pks* gene. Overall, its characteristics perhaps make it the progenitor of a long series of possible future *E. coli* strains capable of creating competition in the host with those indigenous *pks*-positive *E. coli* strains, for which a significant correlation between a higher incidence of CRC and urinary tract tumors and their geographical presence has been recently demonstrated [40]. It is worth remembering that cancer research suggests that colibactin causes DNA damage early after exposure [157, 158]. High-resolution shotgun metagenomic screening of newborns in the UK has shown that *pks*-island-carrying *E. coli* of the B2 phylotype frequently colonize as early as the first 15 days of life [159]. This observation suggests that colibactin-induced DNA damage may accumulate at a young age, thus contributing to the recently reported increased incidence of early-onset colorectal cancer. Indeed, *pks*-positive CRC occurs mainly in young people [39]. Perhaps an innovative approach should be considered to prevent, above all, neonatal but also adult, exposure to colibactin in order to reduce the risk of developing colibactin-associated cancers.

## Supplementary Information

Below is the link to the electronic supplementary material.

Supplementary file1 (ZIP 544417 KB)

Supplementary file2 (ZIP 12597 KB)

## Data Availability

No datasets were generated or analysed during the current study.
