# Supplementary material for: Selection, Comparative Genomics, and Potential Probiotic Features of Escherichia coli 5C, a pks-Negative Strain Isolated from Healthy Infant Donor Feces
Source: Probiotics Antimicrob Proteins. 2025 Apr 16;18(1):436–56. doi: 10.1007/s12602-025-10522-5 (PMC12999690; doi:10.1007/s12602-025-10522-5)
Supplement: Supplementary file 1 — Supplementary file1 (ZIP 544417 KB) [file 12602_2025_10522_MOESM1_ESM.zip › AAT_5C_8C_Nissle_MLST/02.post-processing/assembly_evaluation/GCF043228065/report.pdf]

Report

	contigs_sel
# contigs (>= 0 bp)	4
# contigs (>= 1000 bp)	4
# contigs (>= 5000 bp)	2
# contigs (>= 10000 bp)	1
# contigs (>= 25000 bp)	1
# contigs (>= 50000 bp)	1
Total length (>= 0 bp)	5064940
Total length (>= 1000 bp)	5064940
Total length (>= 5000 bp)	5057244
Total length (>= 10000 bp)	5051730
Total length (>= 25000 bp)	5051730
Total length (>= 50000 bp)	5051730
# contigs	4
Largest contig	5051730
Total length	5064940
GC (%)	50.57
N50	5051730
N90	5051730
auN	5038566.5
L50	1
L90	1
# N's per 100 kbp	0.00

All statistics are based on contigs of size >= 500 bp, unless otherwise noted (e.g., "# contigs (>= 0 bp)" and "Total length (>= 0 bp)" include all contigs).

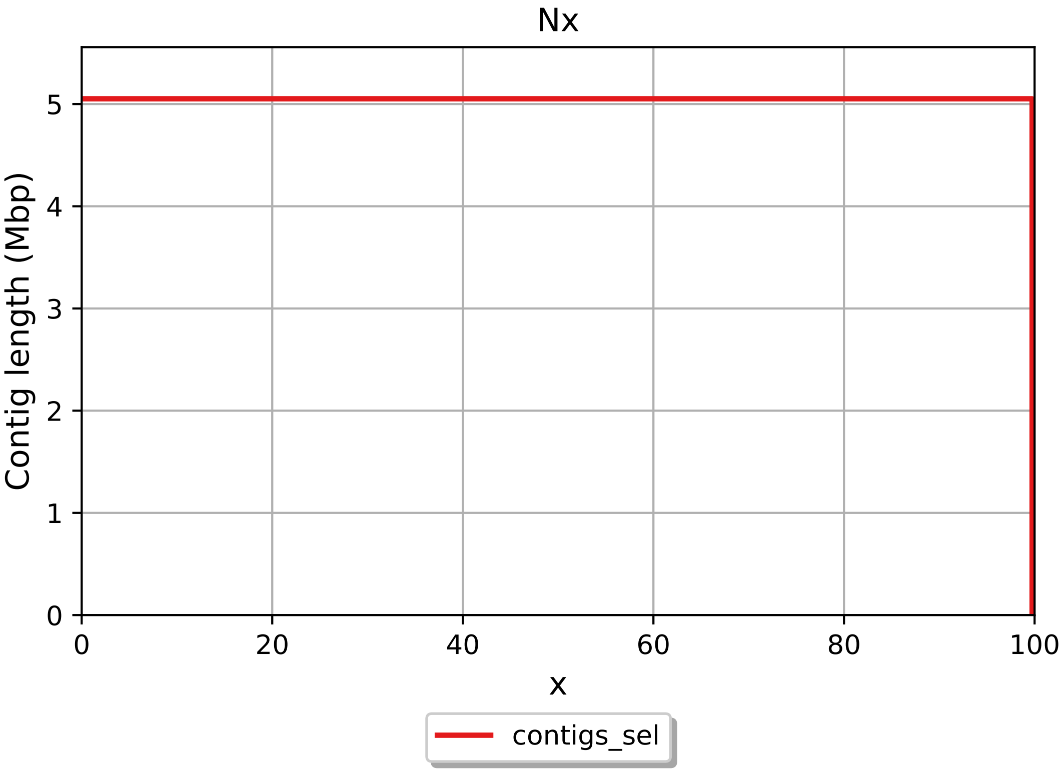
Nx
Contig length (Mbp)
0
1
2
3
4
5
0
20
40
60
80
100
x
contigs_sel

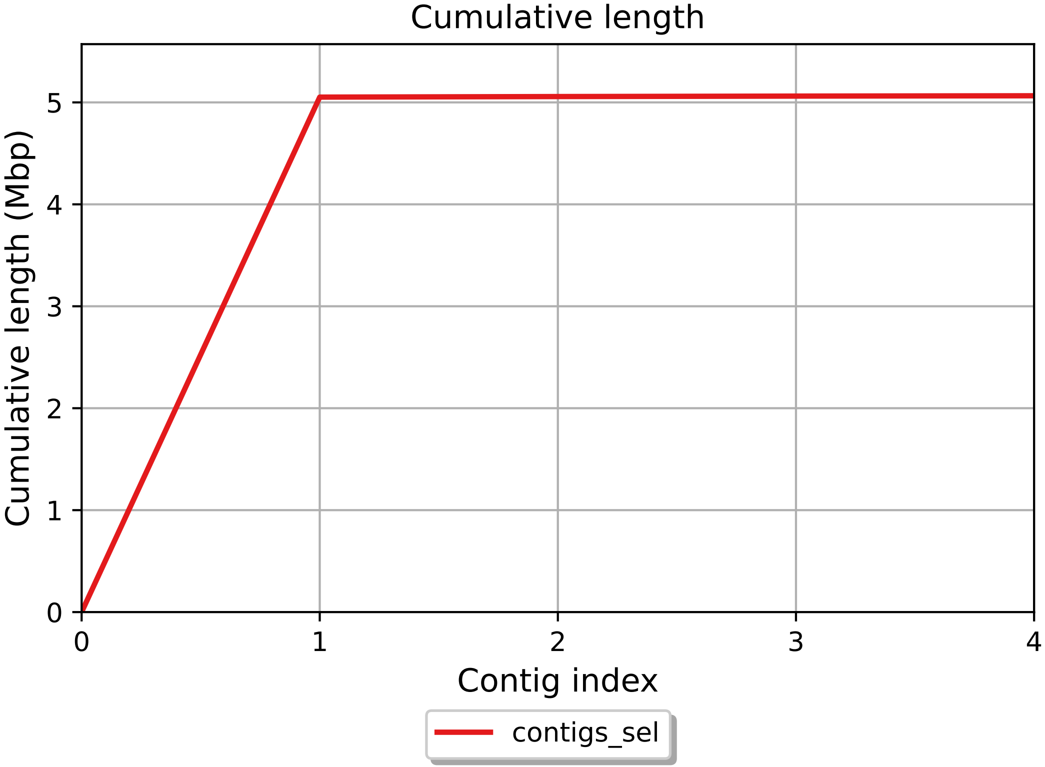
Cumulative length
Cumulative length (Mbp)
0
1
2
3
4
5
0
1
2
3
4
Contig index
contigs_sel

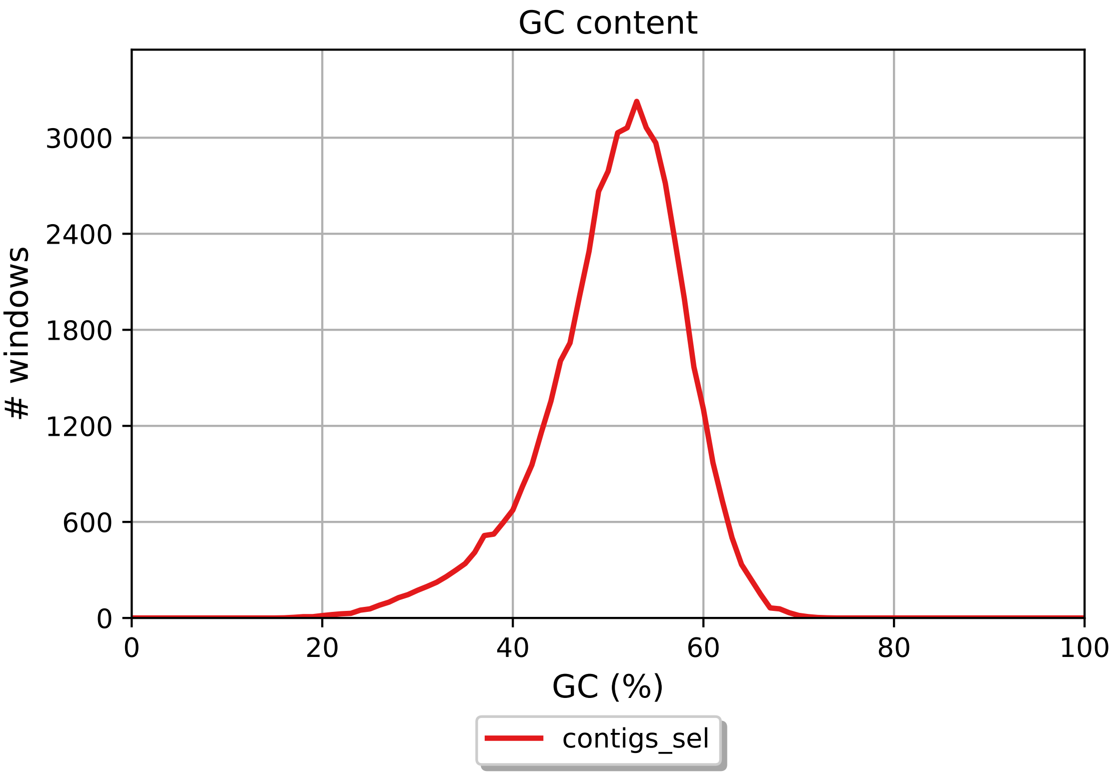
GC content
windows
3000
2400
1800
1200
600
0
0
20
40
60
80
100
GC (%)
contigs_sel

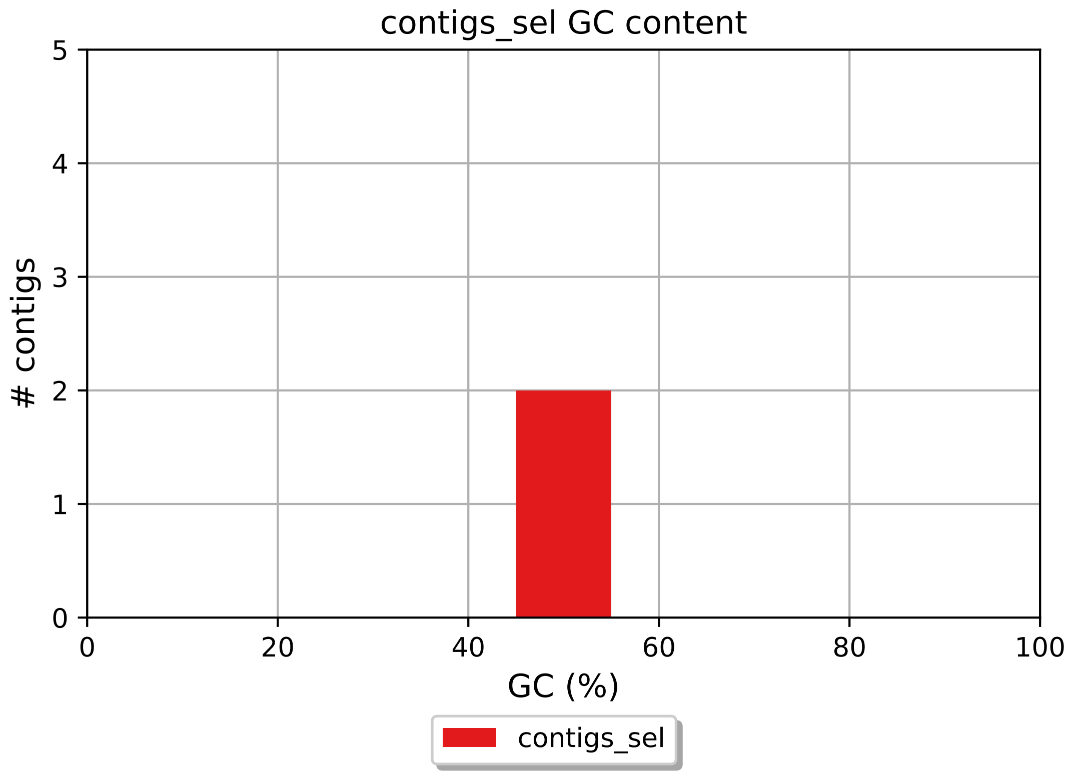
contigs_sel GC content
contigs
GC (%)
0
1
2
3
4
5
0
20
40
60
80
100
contigs_sel
