# Supplementary material for: Selection, Comparative Genomics, and Potential Probiotic Features of Escherichia coli 5C, a pks-Negative Strain Isolated from Healthy Infant Donor Feces
Source: Probiotics Antimicrob Proteins. 2025 Apr 16;18(1):436–56. doi: 10.1007/s12602-025-10522-5 (PMC12999690; doi:10.1007/s12602-025-10522-5)
Supplement: Supplementary file 1 — Supplementary file1 (ZIP 544417 KB) [file 12602_2025_10522_MOESM1_ESM.zip › AAT_5C_8C_Nissle_MLST/02.post-processing/assembly_evaluation/5C/report.pdf]

Report

	contigs_sel
# contigs (>= 0 bp)	40
# contigs (>= 1000 bp)	31
# contigs (>= 5000 bp)	26
# contigs (>= 10000 bp)	25
# contigs (>= 25000 bp)	23
# contigs (>= 50000 bp)	20
Total length (>= 0 bp)	4712575
Total length (>= 1000 bp)	4705612
Total length (>= 5000 bp)	4698762
Total length (>= 10000 bp)	4691055
Total length (>= 25000 bp)	4661266
Total length (>= 50000 bp)	4539862
# contigs	40
Largest contig	509219
Total length	4712575
GC (%)	50.79
N50	335055
N90	91187
auN	302407.1
L50	6
L90	17
# N's per 100 kbp	0.00

All statistics are based on contigs of size >= 500 bp, unless otherwise noted (e.g., "# contigs (>= 0 bp)" and "Total length (>= 0 bp)" include all contigs).

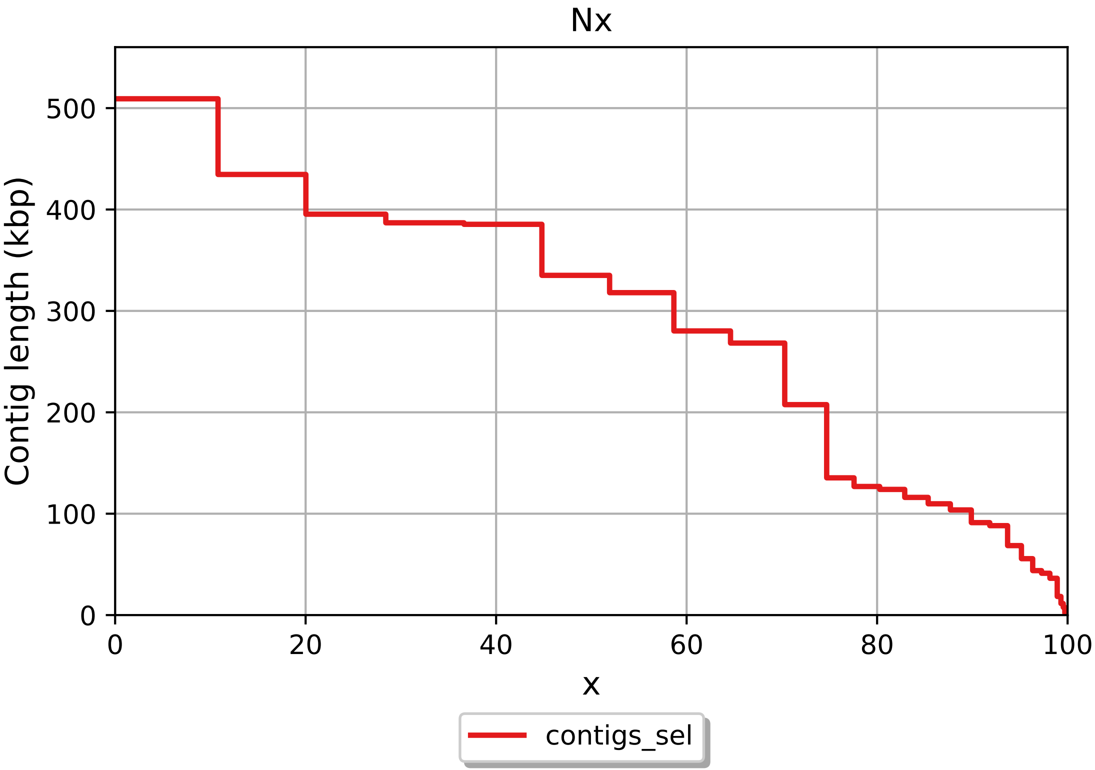
Nx
Contig length (kbp)
500
400
300
200
100
0
0
20
40
60
80
100
x
contigs_sel

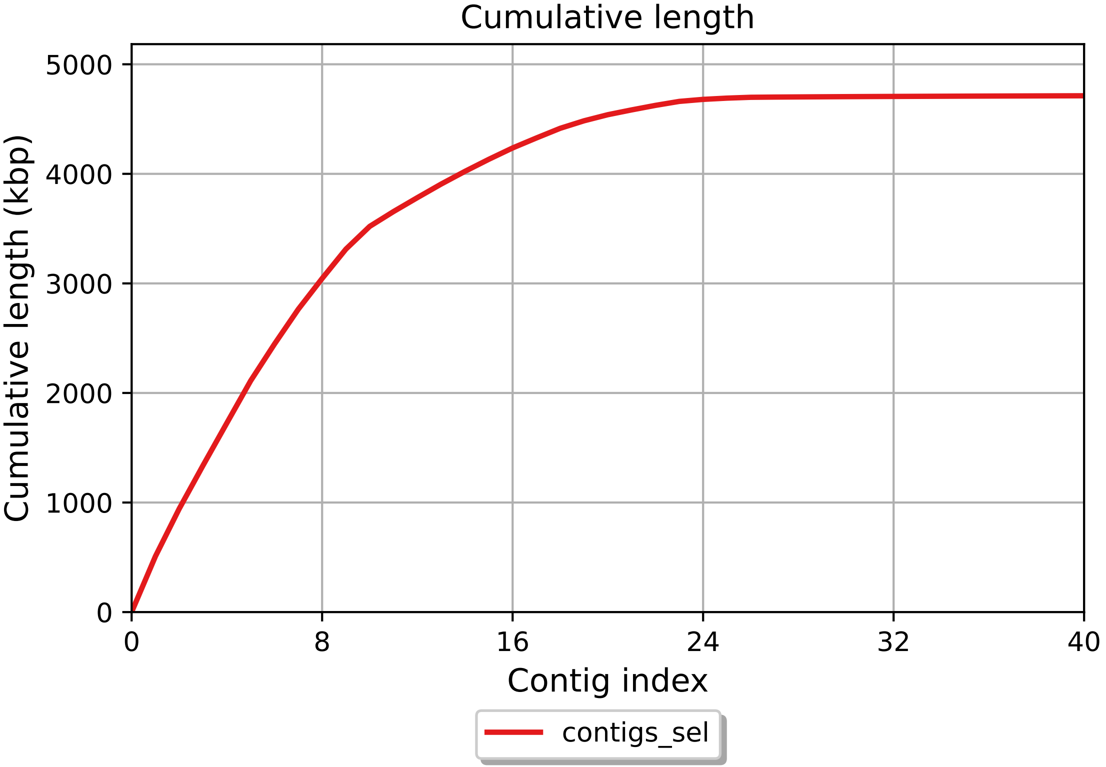

Cumulative length
Cumulative length (kbp)
5000
4000
3000
2000
1000
0
0
8
16
24
32
40
Contig index
contigs_sel

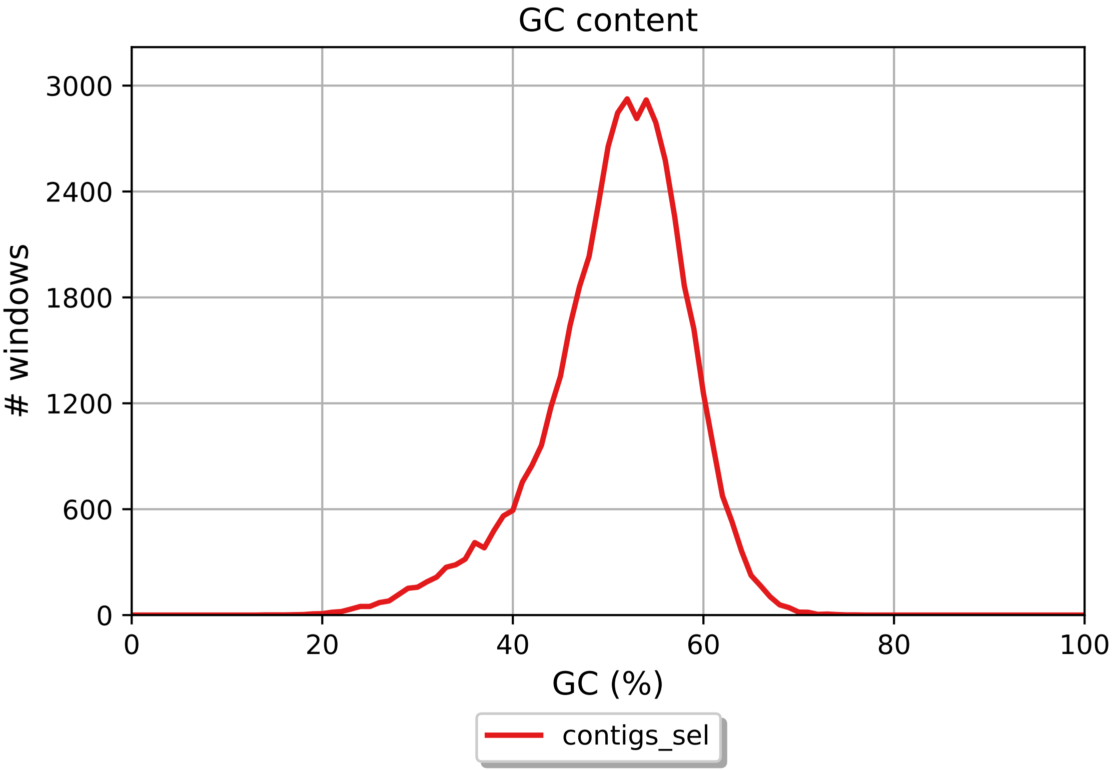

GC content
3000
2400
1800
1200
600
0
windows
0
20
40
60
80
100
GC (%)
contigs_sel

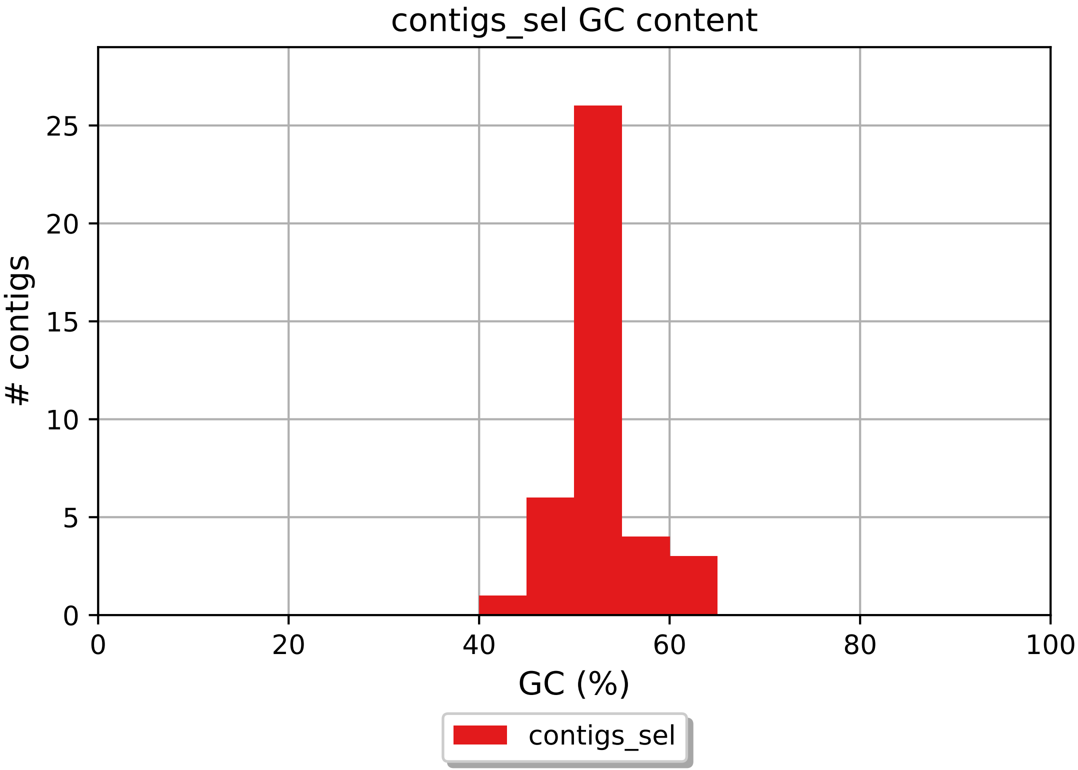
contigs_sel GC content
contigs
0
5
10
15
20
25
0
20
40
60
80
100
GC (%)
contigs_sel

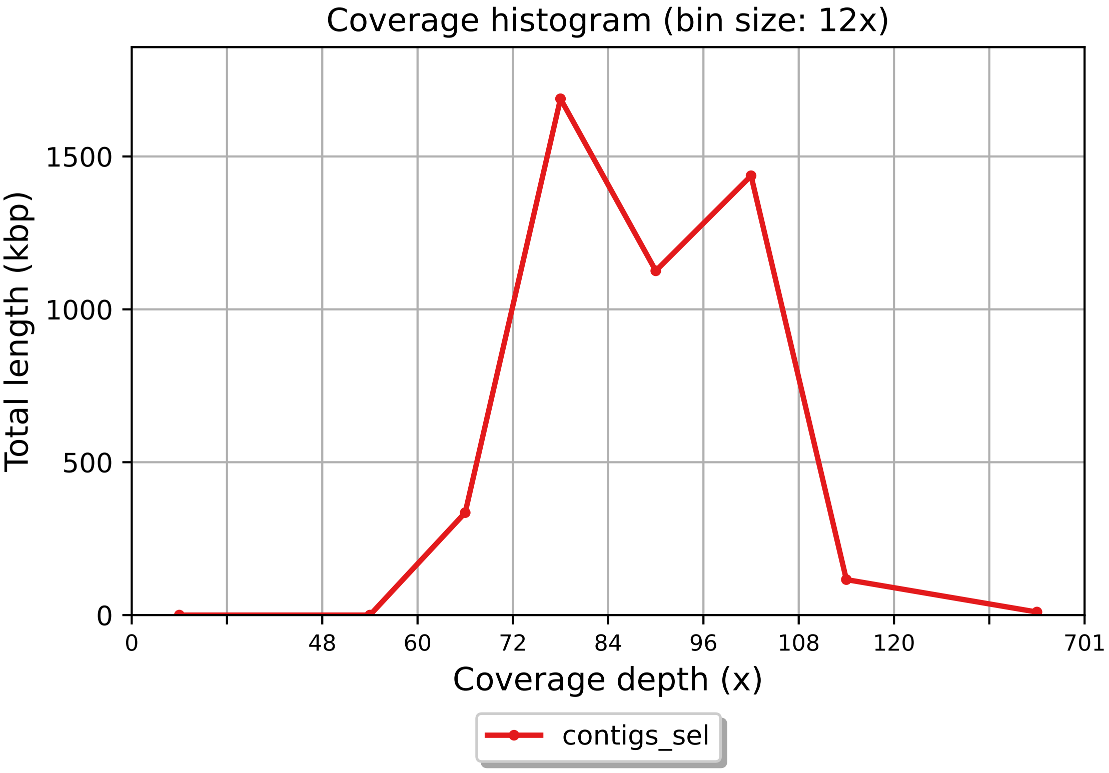

Coverage histogram (bin size: 12x)
Total length (kbp)
0
500
1000
1500
0
48
60
72
84
96
108
120
701
Coverage depth (x)
contigs_sel

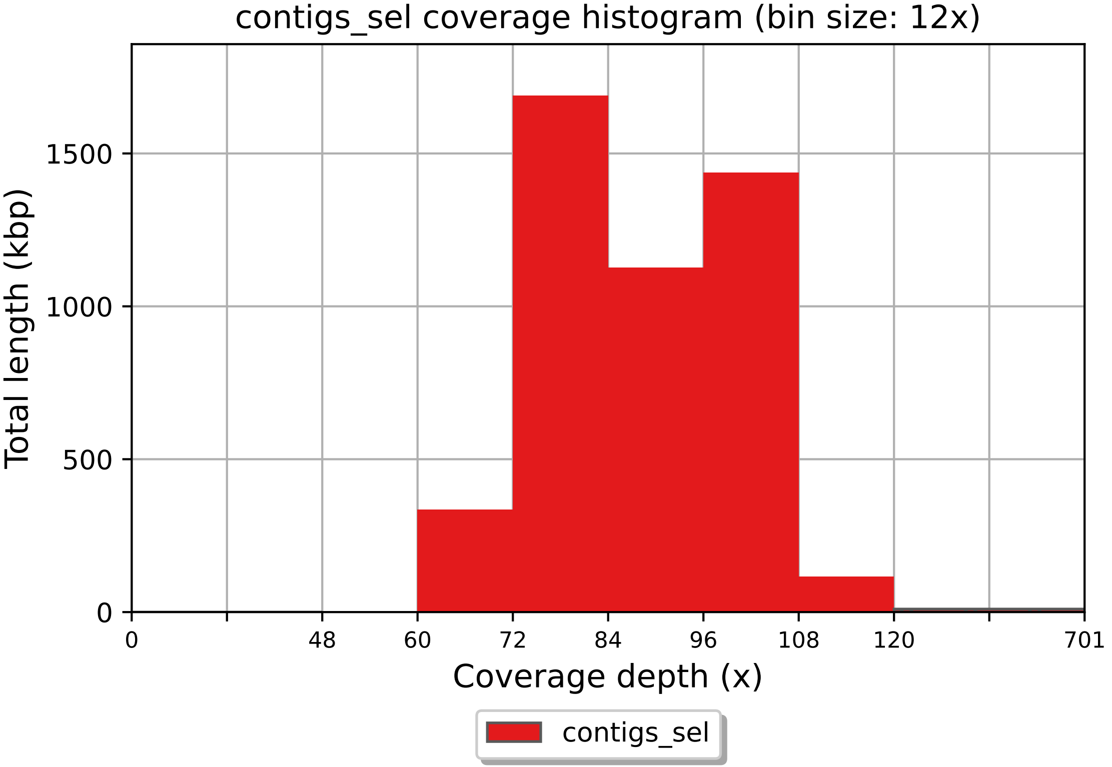
contigs_sel coverage histogram (bin size: 12x)
Total length (kbp)
0
500
1000
1500
0
48
60
72
84
96
108
120
701
Coverage depth (x)
contigs_sel
