# Supplementary material for: Selection, Comparative Genomics, and Potential Probiotic Features of Escherichia coli 5C, a pks-Negative Strain Isolated from Healthy Infant Donor Feces
Source: Probiotics Antimicrob Proteins. 2025 Apr 16;18(1):436–56. doi: 10.1007/s12602-025-10522-5 (PMC12999690; doi:10.1007/s12602-025-10522-5)
Supplement: Supplementary file 1 — Supplementary file1 (ZIP 544417 KB) [file 12602_2025_10522_MOESM1_ESM.zip › AAT_5C_8C_Nissle_MLST/02.post-processing/assembly_evaluation/8C/report.pdf]

Report

	contigs_sel
# contigs (>= 0 bp)	81
# contigs (>= 1000 bp)	62
# contigs (>= 5000 bp)	48
# contigs (>= 10000 bp)	43
# contigs (>= 25000 bp)	36
# contigs (>= 50000 bp)	31
Total length (>= 0 bp)	4929823
Total length (>= 1000 bp)	4917171
Total length (>= 5000 bp)	4890057
Total length (>= 10000 bp)	4855344
Total length (>= 25000 bp)	4725193
Total length (>= 50000 bp)	4562741
# contigs	81
Largest contig	440212
Total length	4929823
GC (%)	50.58
N50	160507
N90	64748
auN	183017.6
L50	10
L90	29
# N's per 100 kbp	0.00

All statistics are based on contigs of size >= 500 bp, unless otherwise noted (e.g., "# contigs (>= 0 bp)" and "Total length (>= 0 bp)" include all contigs).

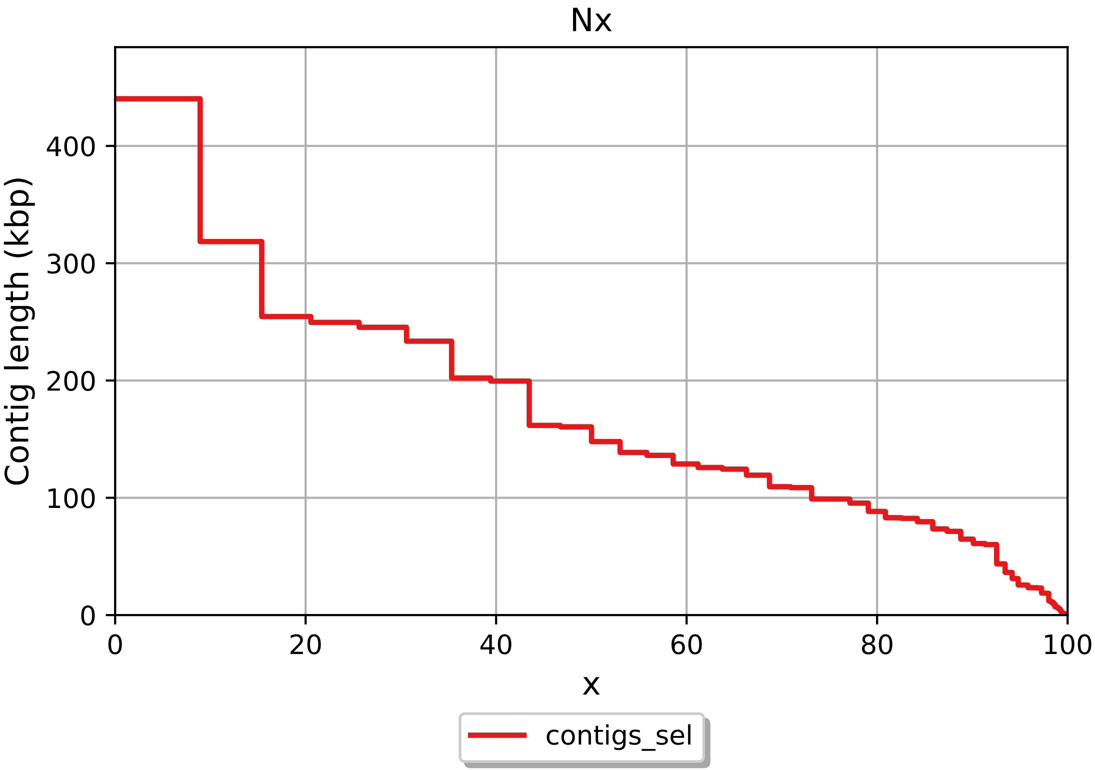

Nx
Contig length (kbp)
0
100
200
300
400
0
20
40
60
80
100
x
contigs_sel

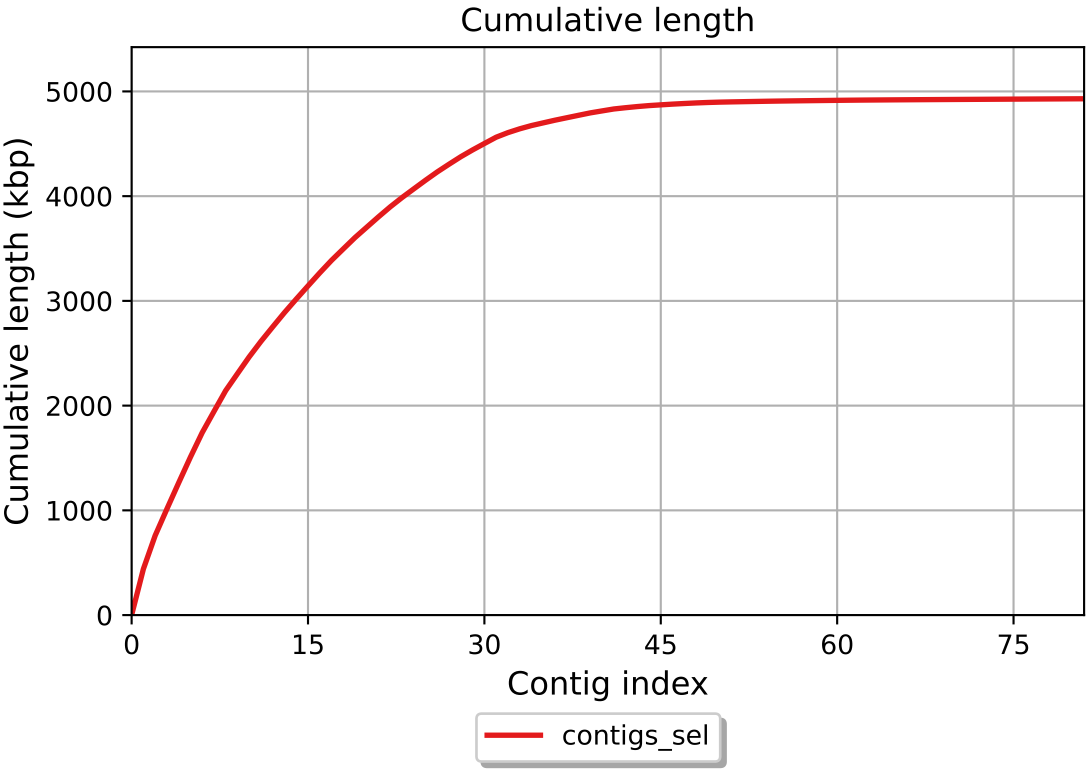

Cumulative length
Cumulative length (kbp)
0
1000
2000
3000
4000
5000
0
15
30
45
60
75
Contig index
contigs_sel

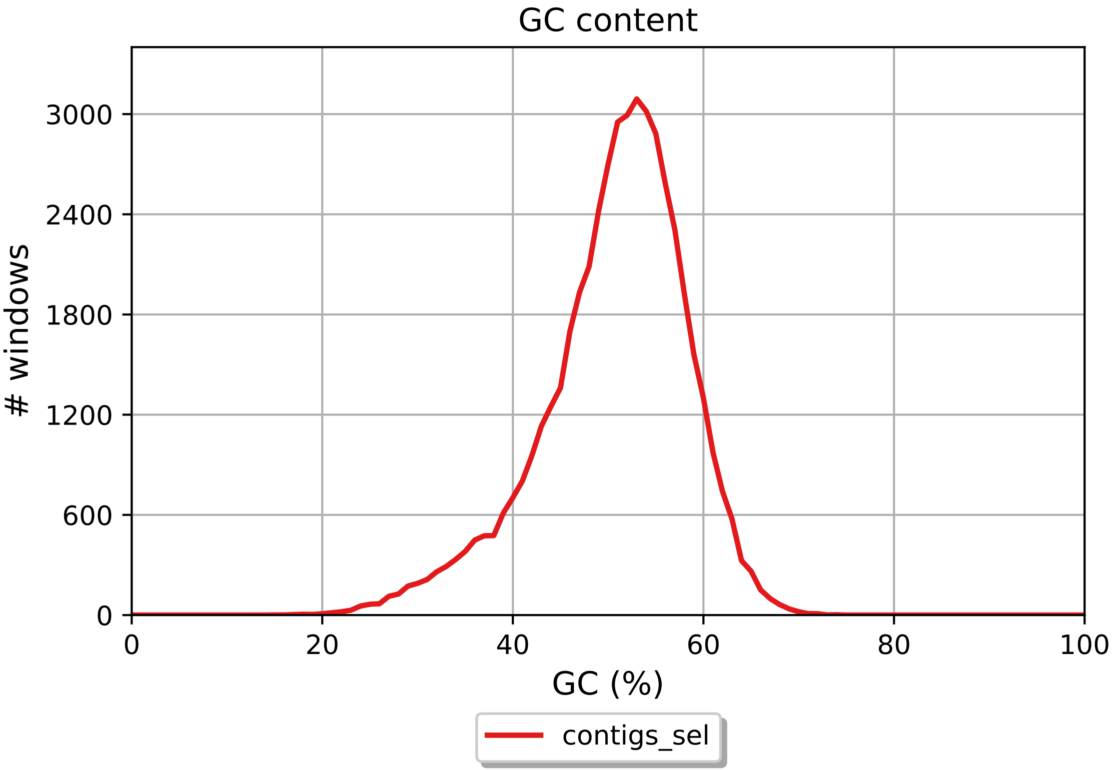

GC content
windows
3000
2400
1800
1200
600
0
0
20
40
60
80
100
GC (%)
contigs_sel

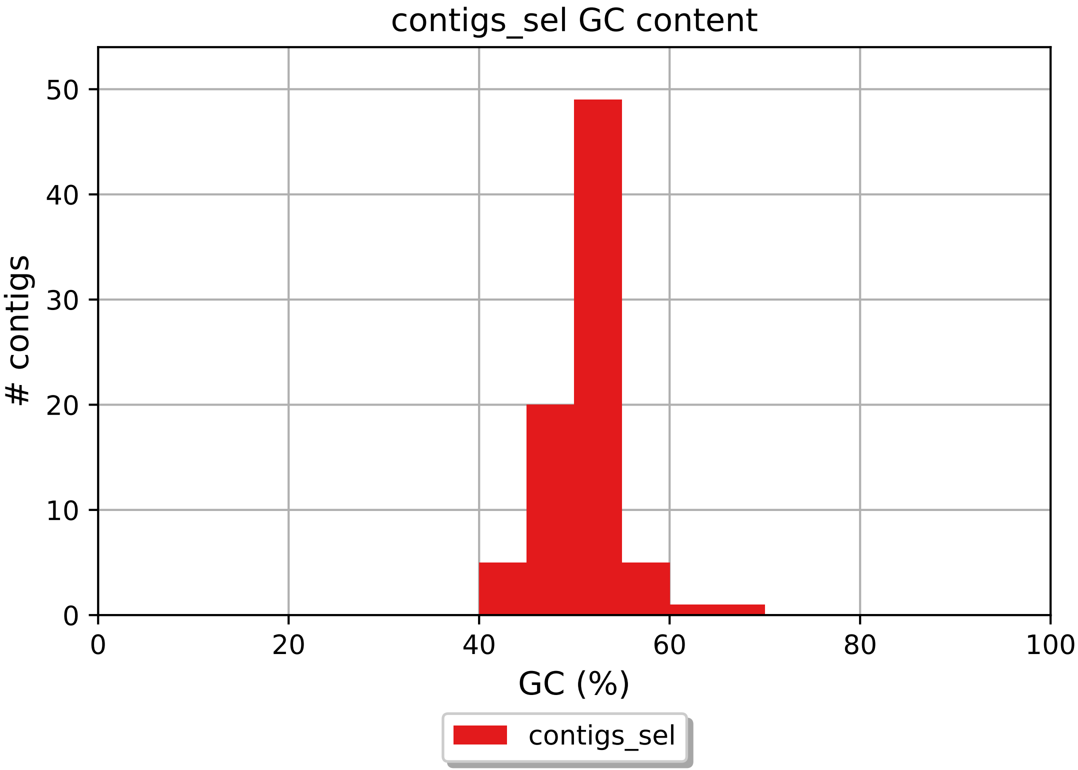

contigs_sel GC content
contigs
0
10
20
30
40
50
0
20
40
60
80
100
GC (%)
contigs_sel

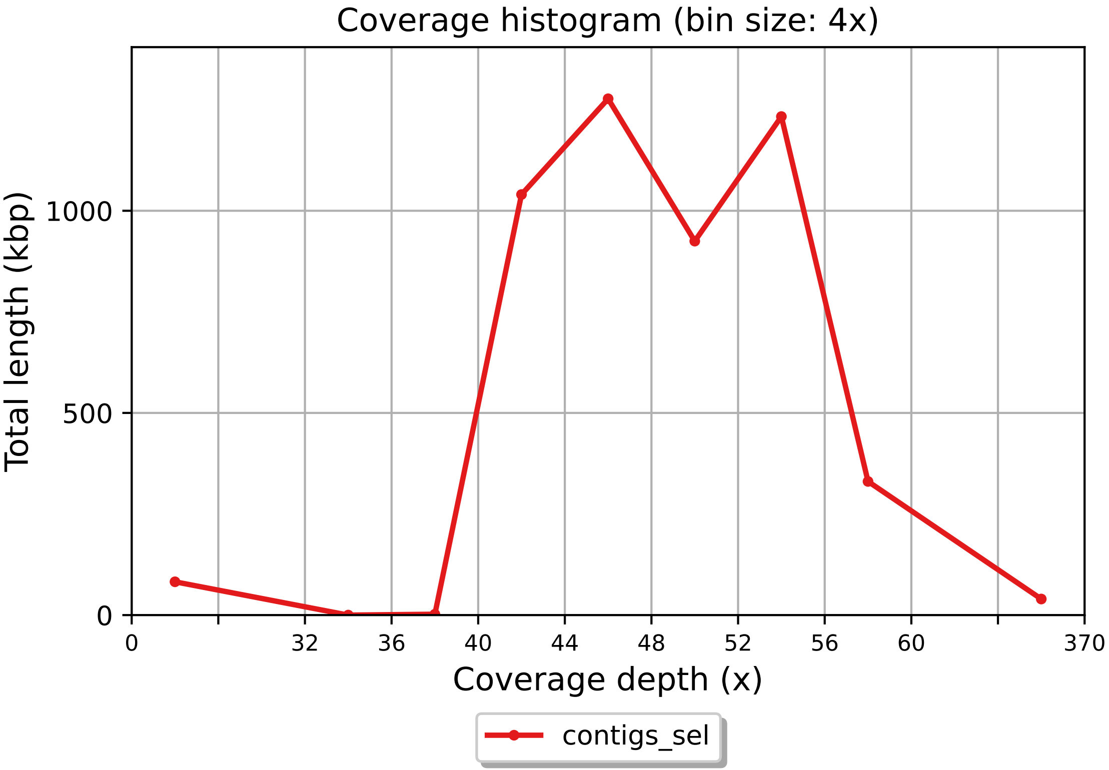

Coverage histogram (bin size: 4x)
Total length (kbp)
0
500
1000
0
32
36
40
44
48
52
56
60
370
Coverage depth (x)
contigs_sel

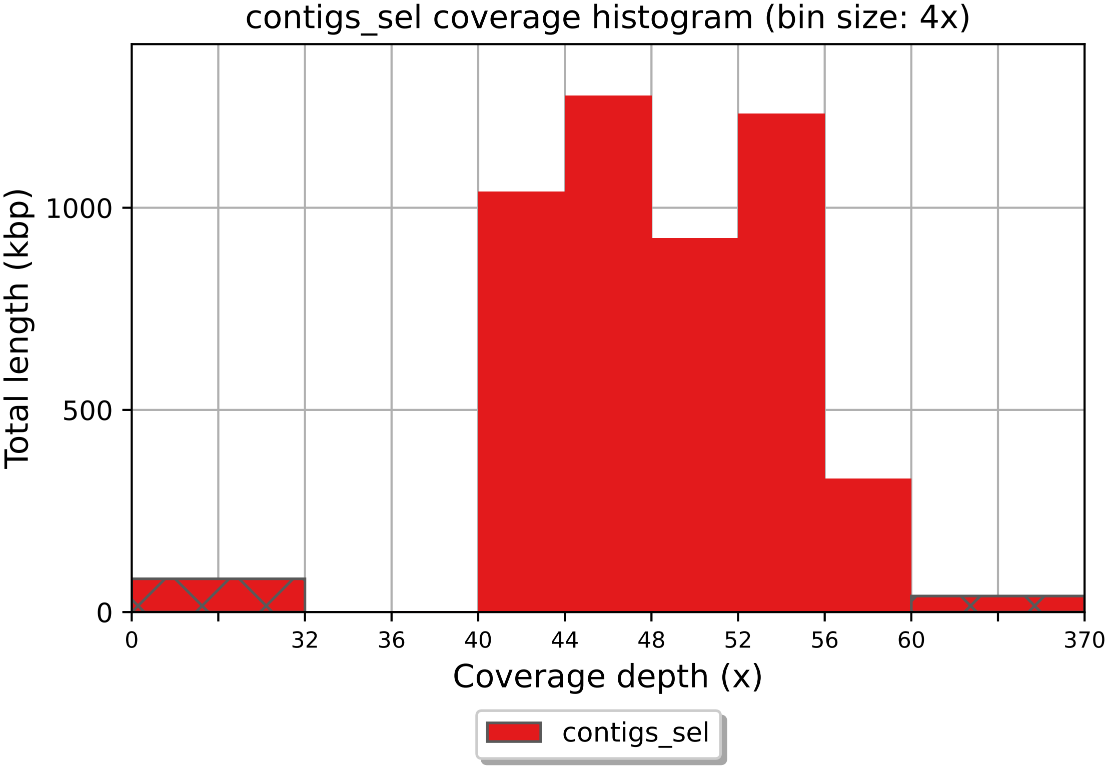
contigs_sel coverage histogram (bin size: 4x)
Total length (kbp)
1000
500
0
0
32
36
40
44
48
52
56
60
370
Coverage depth (x)
contigs_sel
