# Supplementary material for: Selection, Comparative Genomics, and Potential Probiotic Features of Escherichia coli 5C, a pks-Negative Strain Isolated from Healthy Infant Donor Feces
Source: Probiotics Antimicrob Proteins. 2025 Apr 16;18(1):436–56. doi: 10.1007/s12602-025-10522-5 (PMC12999690; doi:10.1007/s12602-025-10522-5)
Supplement: Supplementary file 1 — Supplementary file1 (ZIP 544417 KB) [file 12602_2025_10522_MOESM1_ESM.zip › AAT_5C_8C_Nissle_MLST/04.annotation/antismash/GCF003546975/index.html]

GCF003546975 - 5 region(s) - antiSMASH results


antiSMASH version 7.1.0

Download

- Download all results
- Download GenBank summary file
- Download JSON results file

About

Help

Contact

Select genomic region:

Overview

1.1

1.2

1.3

1.4

1.5

### Identified secondary metabolite regions using strictness 'relaxed'

**contig\_1** (Escherichia sp. GCF003546975)

| Region | Type | From | To | Most similar known cluster | | Similarity |
| --- | --- | --- | --- | --- | --- | --- |
| Region&nbsp1.1 | NRP-metallophore,NRPS,T1PKS | 562,768 | 696,348 |  | | |
| Region&nbsp1.2 | NI-siderophore | 1,731,951 | 1,764,365 |  | | |
| Region&nbsp1.3 | NRP-metallophore,NRPS | 4,121,109 | 4,176,094 |  | | |
| Region&nbsp1.4 | thiopeptide | 4,441,891 | 4,468,183 |  | | |
| Region&nbsp1.5 | arylpolyene | 4,574,316 | 4,617,903 |  | | |

| Region | Type | From | To | Most similar known cluster | | Similarity |
| --- | --- | --- | --- | --- | --- | --- |
| Region&nbsp1.1 | NRP-metallophore,NRPS,T1PKS | 562,768 | 696,348 |  | | |
| Region&nbsp1.2 | NI-siderophore | 1,731,951 | 1,764,365 |  | | |
| Region&nbsp1.3 | NRP-metallophore,NRPS | 4,121,109 | 4,176,094 |  | | |
| Region&nbsp1.4 | thiopeptide | 4,441,891 | 4,468,183 |  | | |
| Region&nbsp1.5 | arylpolyene | 4,574,316 | 4,617,903 |  | | |

No secondary metabolite regions were found in these records:
:   **contig\_2** (Escherichia sp. GCF003546975)
:   **contig\_3** (Escherichia sp. GCF003546975)

Compact view

contig\_1 - Region 1 - NRP-metallophore,NRPS,T1PKS

Shows the layout of the region, marking coding sequences and areas of interest. Clicking a gene will select it and show any relevant details. Clicking an area feature (e.g. a candidate cluster) will select all coding sequences within that area. Double clicking an area feature will zoom to that area. Multiple genes and area features can be selected by clicking them while holding the Ctrl key.  
More detailed help is available here.

Download region GenBank file

Download region SVG

Location: 562,768 - 696,348 nt. (total: 133,581 nt)
Show pHMM detection rules used

NRP-metallophore: (VibH\_like or Cy\_tandem or (cds(Condensation and AMP-binding) and ((IBH\_Asp and not SBH\_Asp) or IBH\_His or TBH\_Asp or CyanoBH\_Asp1 or CyanoBH\_Asp2 or IPL or SalSyn or (EntA and EntC) or (GrbD and GrbE) or (FbnL and FbnM) or PvdO or PvdP or (Orn\_monoox and not (KtzT or MetRS-like)) or Lys\_monoox or VbsL)))  
NRPS: cds(Condensation and (AMP-binding or A-OX))  
T1PKS: cds(PKS\_AT and (PKS\_KS or ene\_KS or mod\_KS or hyb\_KS or itr\_KS or tra\_KS))

#### Legend:

core biosynthetic genes

additional biosynthetic genes

transport-related genes

regulatory genes

other genes

resistance

reset view

zoom to selection

Gene details

Shows details of the most recently selected gene, including names, products, location, and other annotations.

Select a gene to view the details available for it

Gene overview

NRPS/PKS domains

NRPS/PKS modules

Gene/CDS overview

A brief tabular summary of genes/CDS features within the region.  
Filtering the table will also search biosynthetic profiles and gene function data. If enabled, the overview will then zoom to show the area covered by the filtered selection.  
Genes selected in the region drawing above will be marked in the table with an indicator to the left of the gene name.

Filter:

Automatically zoom to filtered/selected features

| Identifier | Product | Length | | Function | Sequence | | NCBI Blast | Filter details |
| --- | --- | --- | --- | --- | --- | --- | --- | --- |
|  |  | NT | AA |  | NT | AA |  |  |

No genes match the given filter

Detailed domain annotation

Shows NRPS- and PKS-related domains for each feature that contains them. Click on each domain for more information about the domain's location, consensus monomer prediction, and other details.  
A domain glossary is available here, and an explanation of the visualisation is available here.

Selected features only

Show module domains

Module view

Shows module structures for each candidate cluster in NRPS and PKS regions.   
Genes are shown in predicted order, and are only present when containing at least one complete module.   
A domain glossary is available here, and an explanation of the visualisation is available here.

Candidate 1 (562767 - 696348): neighbouring NRP-metallophore-NRPS-T1PKS

Candidate 2 (562767 - 616388): chemical hybrid NRP-metallophore-NRPS

Candidate 3 (576475 - 625967): single T1PKS

Candidate 4 (612264 - 696348): chemical hybrid NRPS-T1PKS

Legend

NRPS/PKS products

NRPS/PKS substrates

Predicted core structure(s)

Shows estimated product structure and polymer for each candidate cluster in the region. To show the product, click on the expander or the candidate cluster feature drawn in the overview.

For candidate cluster 1, location 562767 - 696348:

Rough prediction of core scaffold based on assumed PKS/NRPS colinearity; tailoring reactions not taken into account

**Polymer prediction:**
:   (Ala - redmal) + (X) + (mal) + (Gly - Cys) + (Cys) + (Asn) + (Me-ohmal) + (Cys)

  
Direct lookup in NORINE database:
strict
or
relaxed

---

For candidate cluster 2, location 562767 - 616388:

Rough prediction of core scaffold based on assumed PKS/NRPS colinearity; tailoring reactions not taken into account

**Polymer prediction:**
:   (Cys) + (Me-ohmal)

  
Direct lookup in NORINE database:
strict
or
relaxed

---

For candidate cluster 3, location 576475 - 625967:

Rough prediction of core scaffold based on assumed PKS/NRPS colinearity; tailoring reactions not taken into account

**Polymer prediction:**
:   (Cys) + (Me-ohmal)

  
Direct lookup in NORINE database:
strict
or
relaxed

---

For candidate cluster 4, location 612264 - 696348:

Rough prediction of core scaffold based on assumed PKS/NRPS colinearity; tailoring reactions not taken into account

**Polymer prediction:**
:   (Ala - redmal) + (X) + (mal) + (Gly - Cys) + (Cys) + (Asn)

  
Direct lookup in NORINE database:
strict
or
relaxed

Link to NORINE database query form

NRPS/PKS substrate predictions

Shows the predicted substrates for each adenylation domain and acyltransferase within genes. Each gene prediction can be expanded to view detailed predictions of each domain. Each prediction can be expanded to view the predictions by tool (and, for some tools, further expanded for extra details).

**GCF003546975\_03065**: Cys

:   Search NORINE for peptide:
    strict
    or
    relaxed
  
:   **AMP-binding (573..966)**: Cys

    nrpys: Cys

    SVM prediction details:
    :   Predicted physicochemical class:
        :   hydrophobic-aliphatic (Ala, Gly, Val, Leu, Ile, Abu, Ival, Ser, Thr, Hpg, Dhpg, Cys, Pro, Hpr)

        Large clusters prediction:
        :   Polar, uncharged (aliphatic with -SH) (Cys)

        Small clusters prediction:
        :   Polar, uncharged (aliphatic with -SH) (Cys)

        Single AA prediction:
        :   Polar, uncharged (aliphatic with -SH) (Cys)

    Stachelhaus prediction details:
    :   Stachelhaus sequence:
        :   DLYNMSMIWK

        Nearest Stachelhaus code(s):
        :   Cys DLYNMSMIWK (100% 8Å match)

        Stachelhaus code match:
        :   100% (strong)

  
**GCF003546975\_03070**: mal

:   **PKS\_AT (552..842)**: mal

    ATSignature: Malonyl-CoA

    Top 3 matches:
    :   Malonyl-CoA: 100.0%
    :   inactive: 62.5%
    :   2-Rhyd-Malonyl-CoA: 58.3%

      
    minowa: Malonyl-CoA

    Prediction, score:
    :   Malonyl-CoA: 132.8


        Methoxymalonyl-CoA: 50.0


        inactive: 45.6


        Methylmalonyl-CoA: 44.0


        Benzoyl-CoA: 42.7


        Propionyl-CoA: 34.5


        Isobutyryl-CoA: 31.1


        fatty\_acid: 25.6


        Acetyl-CoA: 25.3


        2-Methylbutyryl-CoA: 24.6


        3-Methylbutyryl-CoA: 19.5


        CHC-CoA: 16.3


        trans-1,2-CPDA: 11.2


        Ethylmalonyl-CoA: 7.1

  
**GCF003546975\_03085**: diOH-Bz

:   Search NORINE for peptide:
    strict
    or
    relaxed
  
:   **AMP-binding (20..422)**: diOH-Bz

    nrpys: diOH-Bz

    SVM prediction details:
    :   Predicted physicochemical class:
        :   hydrophobic-aromatic (Phe, Tyr, diOH-Bz, Ph-Gly, bOH-Tyr)

        Large clusters prediction:
        :   Hydroxy-benzoic acid derivates (diOH-Bz, Sal)

        Small clusters prediction:
        :   Hydroxy-benzoic acid derivates (diOH-Bz, Sal)

        Single AA prediction:
        :   dhb (diOH-Bz)

    Stachelhaus prediction details:
    :   Stachelhaus sequence:
        :   PLPAQGVLCK

        Nearest Stachelhaus code(s):
        :   diOH-Bz PLPAQGVLCK (100% 8Å match)

        Stachelhaus code match:
        :   100% (strong)

  
**GCF003546975\_03185**: Asn

:   Search NORINE for peptide:
    strict
    or
    relaxed
  
:   **AMP-binding (453..844)**: Asn

    nrpys: Asn

    SVM prediction details:
    :   Predicted physicochemical class:
        :   hydrophilic (Arg, Asp, Glu, Asn, Lys, Gln, Orn, Aad)

        Large clusters prediction:
        :   Aliphatic chain with H-bond donor (Asp, Asn, Glu, Gln, Aad)

        Small clusters prediction:
        :   asp,asn (Asp, Asn)

        Single AA prediction:
        :   asn (Asn)

    Stachelhaus prediction details:
    :   Stachelhaus sequence:
        :   DLTKVGEVGK

        Nearest Stachelhaus code(s):
        :   Asn DLTKVGEVGK (100% 8Å match)

        Stachelhaus code match:
        :   100% (strong)

  
**GCF003546975\_03200**: Cys

:   Search NORINE for peptide:
    strict
    or
    relaxed
  
:   **AMP-binding (1269..1664)**: Cys

    nrpys: Cys

    SVM prediction details:
    :   Predicted physicochemical class:
        :   hydrophobic-aliphatic (Ala, Gly, Val, Leu, Ile, Abu, Ival, Ser, Thr, Hpg, Dhpg, Cys, Pro, Hpr)

        Large clusters prediction:
        :   Polar, uncharged (aliphatic with -SH) (Cys)

        Small clusters prediction:
        :   Polar, uncharged (aliphatic with -SH) (Cys)

        Single AA prediction:
        :   Polar, uncharged (aliphatic with -SH) (Cys)

    Stachelhaus prediction details:
    :   Stachelhaus sequence:
        :   DLYNLSLIWK

        Nearest Stachelhaus code(s):
        :   Cys DLYNLSLIWK (100% 8Å match)

        Stachelhaus code match:
        :   100% (strong)

  
**GCF003546975\_03205**: Gly - Cys

:   Search NORINE for peptide:
    strict
    or
    relaxed
  
:   **AMP-binding (507..894)**: Gly

    nrpys: Gly

    SVM prediction details:
    :   Predicted physicochemical class:
        :   hydrophobic-aliphatic (Ala, Gly, Val, Leu, Ile, Abu, Ival, Ser, Thr, Hpg, Dhpg, Cys, Pro, Hpr)

        Large clusters prediction:
        :   Apolar, aliphatic (Gly, Ala, Val, Leu, Ile, Abu, Ival)

        Small clusters prediction:
        :   Tiny, hydrophilic, transition to aliphatic (Gly, Ala)

        Single AA prediction:
        :   gly (Gly)

    Stachelhaus prediction details:
    :   Stachelhaus sequence:
        :   DILQLGLIWK

        Nearest Stachelhaus code(s):
        :   Gly DILQLGLIWK (100% 8Å match)

        Stachelhaus code match:
        :   100% (strong)
:   **AMP-binding (1555..1950)**: Cys

    nrpys: Cys

    SVM prediction details:
    :   Predicted physicochemical class:
        :   hydrophobic-aliphatic (Ala, Gly, Val, Leu, Ile, Abu, Ival, Ser, Thr, Hpg, Dhpg, Cys, Pro, Hpr)

        Large clusters prediction:
        :   Polar, uncharged (aliphatic with -SH) (Cys)

        Small clusters prediction:
        :   Polar, uncharged (aliphatic with -SH) (Cys)

        Single AA prediction:
        :   Polar, uncharged (aliphatic with -SH) (Cys)

    Stachelhaus prediction details:
    :   Stachelhaus sequence:
        :   DLYNLSLIWK

        Nearest Stachelhaus code(s):
        :   Cys DLYNLSLIWK (100% 8Å match)

        Stachelhaus code match:
        :   100% (strong)

  
**GCF003546975\_03210**: mal

:   **PKS\_AT (531..813)**: mal

    ATSignature: Malonyl-CoA

    Top 3 matches:
    :   Malonyl-CoA: 87.5%
    :   inactive: 79.2%
    :   Propionyl-CoA: 54.2%

      
    minowa: Malonyl-CoA

    Prediction, score:
    :   Malonyl-CoA: 135.2


        inactive: 78.0


        Methoxymalonyl-CoA: 66.0


        Methylmalonyl-CoA: 56.3


        Isobutyryl-CoA: 28.5


        Propionyl-CoA: 26.3


        Ethylmalonyl-CoA: 20.5


        2-Methylbutyryl-CoA: 14.2


        Benzoyl-CoA: 11.6


        trans-1,2-CPDA: 11.4


        CHC-CoA: 9.8


        3-Methylbutyryl-CoA: 6.9


        fatty\_acid: 0.0


        Acetyl-CoA: 0.0

  
**GCF003546975\_03215**: Ser - X

:   Search NORINE for peptide:
    strict
    or
    relaxed
  
:   **AMP-binding (24..422)**: Ser

    nrpys: Ser

    SVM prediction details:
    :   Predicted physicochemical class:
        :   hydrophobic-aliphatic (Ala, Gly, Val, Leu, Ile, Abu, Ival, Ser, Thr, Hpg, Dhpg, Cys, Pro, Hpr)

        Large clusters prediction:
        :   Aliphatic chain or phenyl group with -OH (Ser, Thr, Dhpg, Hpg)

        Small clusters prediction:
        :   ser (Ser)

        Single AA prediction:
        :   ser (Ser)

    Stachelhaus prediction details:
    :   Stachelhaus sequence:
        :   DVWHFSLIDK

        Nearest Stachelhaus code(s):
        :   Ser DVWHFSLIDK (100% 8Å match)

        Stachelhaus code match:
        :   100% (strong)
:   **AMP-binding (992..1384)**: X

    nrpys: Sam

    SVM prediction details:
    :   Predicted physicochemical class:
        :   hydrophobic-aromatic (Phe, Tyr, diOH-Bz, Ph-Gly, bOH-Tyr)

        Large clusters prediction:
        :   Aromatic side chain (Phe, Trp, Ph-Gly, Tyr, bOH-Tyr)

        Small clusters prediction:
        :   N/A

        Single AA prediction:
        :   N/A

    Stachelhaus prediction details:
    :   Stachelhaus sequence:
        :   ASGSLGGICK

        Nearest Stachelhaus code(s):
        :   Sam ASGSLGGICK (100% 8Å match)

        Stachelhaus code match:
        :   100% (strong)

  
**GCF003546975\_03220**: mal

:   **PKS\_AT (7..296)**: mal

    ATSignature: Malonyl-CoA

    Top 3 matches:
    :   Malonyl-CoA: 58.3%

      
    minowa: Malonyl-CoA

    Prediction, score:
    :   Malonyl-CoA: 57.2


        Methoxymalonyl-CoA: 41.1


        Methylmalonyl-CoA: 34.3


        inactive: 30.1


        Ethylmalonyl-CoA: 18.1


        Propionyl-CoA: 16.0


        Acetyl-CoA: 14.6


        Benzoyl-CoA: 12.9


        2-Methylbutyryl-CoA: 12.5


        CHC-CoA: 9.1


        trans-1,2-CPDA: 0.0


        fatty\_acid: 0.0


        Isobutyryl-CoA: 0.0


        3-Methylbutyryl-CoA: 0.0

  
**GCF003546975\_03245**: Ala - mal

:   Search NORINE for peptide:
    strict
    or
    relaxed
  
:   **AMP-binding (502..890)**: Ala

    nrpys: Ala

    SVM prediction details:
    :   Predicted physicochemical class:
        :   hydrophobic-aliphatic (Ala, Gly, Val, Leu, Ile, Abu, Ival, Ser, Thr, Hpg, Dhpg, Cys, Pro, Hpr)

        Large clusters prediction:
        :   Apolar, aliphatic (Gly, Ala, Val, Leu, Ile, Abu, Ival)

        Small clusters prediction:
        :   Aliphatic, branched hydrophobic (Val, Leu, Ile, Abu, Ival)

        Single AA prediction:
        :   val (Val)

    Stachelhaus prediction details:
    :   Stachelhaus sequence:
        :   DVFWTGGIFK

        Nearest Stachelhaus code(s):
        :   Ala DVFWTGGIFK (100% 8Å match)

        Stachelhaus code match:
        :   100% (strong)
:   **PKS\_AT (1604..1895)**: mal

    ATSignature: Malonyl-CoA

    Top 3 matches:
    :   Malonyl-CoA: 87.5%
    :   inactive: 79.2%

      
    minowa: Malonyl-CoA

    Prediction, score:
    :   Malonyl-CoA: 155.9


        inactive: 95.9


        Methoxymalonyl-CoA: 53.4


        Methylmalonyl-CoA: 38.8


        Propionyl-CoA: 26.7


        Isobutyryl-CoA: 21.1


        fatty\_acid: 18.9


        CHC-CoA: 16.9


        2-Methylbutyryl-CoA: 11.0


        Benzoyl-CoA: 10.3


        Ethylmalonyl-CoA: 9.3


        Acetyl-CoA: 8.1


        trans-1,2-CPDA: 0.0


        3-Methylbutyryl-CoA: 0.0

contig\_1 - Region 2 - NI-siderophore

Shows the layout of the region, marking coding sequences and areas of interest. Clicking a gene will select it and show any relevant details. Clicking an area feature (e.g. a candidate cluster) will select all coding sequences within that area. Double clicking an area feature will zoom to that area. Multiple genes and area features can be selected by clicking them while holding the Ctrl key.  
More detailed help is available here.

Download region GenBank file

Download region SVG

Location: 1,731,951 - 1,764,365 nt. (total: 32,415 nt)
Show pHMM detection rules used

NI-siderophore: IucA\_IucC

#### Legend:

core biosynthetic genes

additional biosynthetic genes

transport-related genes

regulatory genes

other genes

resistance

reset view

zoom to selection

Gene details

Shows details of the most recently selected gene, including names, products, location, and other annotations.

Select a gene to view the details available for it

Gene overview

Gene/CDS overview

A brief tabular summary of genes/CDS features within the region.  
Filtering the table will also search biosynthetic profiles and gene function data. If enabled, the overview will then zoom to show the area covered by the filtered selection.  
Genes selected in the region drawing above will be marked in the table with an indicator to the left of the gene name.

Filter:

Automatically zoom to filtered/selected features

| Identifier | Product | Length | | Function | Sequence | | NCBI Blast | Filter details |
| --- | --- | --- | --- | --- | --- | --- | --- | --- |
|  |  | NT | AA |  | NT | AA |  |  |

No genes match the given filter

contig\_1 - Region 3 - NRP-metallophore,NRPS

Shows the layout of the region, marking coding sequences and areas of interest. Clicking a gene will select it and show any relevant details. Clicking an area feature (e.g. a candidate cluster) will select all coding sequences within that area. Double clicking an area feature will zoom to that area. Multiple genes and area features can be selected by clicking them while holding the Ctrl key.  
More detailed help is available here.

Download region GenBank file

Download region SVG

Location: 4,121,109 - 4,176,094 nt. (total: 54,986 nt)
Show pHMM detection rules used

NRP-metallophore: (VibH\_like or Cy\_tandem or (cds(Condensation and AMP-binding) and ((IBH\_Asp and not SBH\_Asp) or IBH\_His or TBH\_Asp or CyanoBH\_Asp1 or CyanoBH\_Asp2 or IPL or SalSyn or (EntA and EntC) or (GrbD and GrbE) or (FbnL and FbnM) or PvdO or PvdP or (Orn\_monoox and not (KtzT or MetRS-like)) or Lys\_monoox or VbsL)))  
NRPS: cds(Condensation and (AMP-binding or A-OX))

#### Legend:

core biosynthetic genes

additional biosynthetic genes

transport-related genes

regulatory genes

other genes

resistance

reset view

zoom to selection

Gene details

Shows details of the most recently selected gene, including names, products, location, and other annotations.

Select a gene to view the details available for it

Gene overview

NRPS/PKS domains

NRPS/PKS modules

Gene/CDS overview

A brief tabular summary of genes/CDS features within the region.  
Filtering the table will also search biosynthetic profiles and gene function data. If enabled, the overview will then zoom to show the area covered by the filtered selection.  
Genes selected in the region drawing above will be marked in the table with an indicator to the left of the gene name.

Filter:

Automatically zoom to filtered/selected features

| Identifier | Product | Length | | Function | Sequence | | NCBI Blast | Filter details |
| --- | --- | --- | --- | --- | --- | --- | --- | --- |
|  |  | NT | AA |  | NT | AA |  |  |

No genes match the given filter

Detailed domain annotation

Shows NRPS- and PKS-related domains for each feature that contains them. Click on each domain for more information about the domain's location, consensus monomer prediction, and other details.  
A domain glossary is available here, and an explanation of the visualisation is available here.

Selected features only

Show module domains

Module view

Shows module structures for each candidate cluster in NRPS and PKS regions.   
Genes are shown in predicted order, and are only present when containing at least one complete module.   
A domain glossary is available here, and an explanation of the visualisation is available here.

Candidate 6 (4121108 - 4176094): chemical hybrid NRP-metallophore-NRPS

Legend

NRPS/PKS products

NRPS/PKS substrates

Predicted core structure(s)

Shows estimated product structure and polymer for each candidate cluster in the region. To show the product, click on the expander or the candidate cluster feature drawn in the overview.

For candidate cluster 6, location 4121108 - 4176094:

Rough prediction of core scaffold based on assumed PKS/NRPS colinearity; tailoring reactions not taken into account

**Polymer prediction:**
:   (Ser)

  
Direct lookup in NORINE database:
strict
or
relaxed

Link to NORINE database query form

NRPS/PKS substrate predictions

Shows the predicted substrates for each adenylation domain and acyltransferase within genes. Each gene prediction can be expanded to view detailed predictions of each domain. Each prediction can be expanded to view the predictions by tool (and, for some tools, further expanded for extra details).

**GCF003546975\_20200**: Ser

:   Search NORINE for peptide:
    strict
    or
    relaxed
  
:   **AMP-binding (461..863)**: Ser

    nrpys: Ser

    SVM prediction details:
    :   Predicted physicochemical class:
        :   hydrophobic-aliphatic (Ala, Gly, Val, Leu, Ile, Abu, Ival, Ser, Thr, Hpg, Dhpg, Cys, Pro, Hpr)

        Large clusters prediction:
        :   Aliphatic chain or phenyl group with -OH (Ser, Thr, Dhpg, Hpg)

        Small clusters prediction:
        :   ser (Ser)

        Single AA prediction:
        :   ser (Ser)

    Stachelhaus prediction details:
    :   Stachelhaus sequence:
        :   DVWHFSLVDK

        Nearest Stachelhaus code(s):
        :   Ser DVWHFSLVDK (100% 8Å match)

        Stachelhaus code match:
        :   100% (strong)

  
**GCF003546975\_20240**: diOH-Bz

:   Search NORINE for peptide:
    strict
    or
    relaxed
  
:   **AMP-binding (34..437)**: diOH-Bz

    nrpys: diOH-Bz

    SVM prediction details:
    :   Predicted physicochemical class:
        :   hydrophobic-aromatic (Phe, Tyr, diOH-Bz, Ph-Gly, bOH-Tyr)

        Large clusters prediction:
        :   Hydroxy-benzoic acid derivates (diOH-Bz, Sal)

        Small clusters prediction:
        :   Hydroxy-benzoic acid derivates (diOH-Bz, Sal)

        Single AA prediction:
        :   dhb (diOH-Bz)

    Stachelhaus prediction details:
    :   Stachelhaus sequence:
        :   AMPAQGVVNK

        Nearest Stachelhaus code(s):
        :   diOH-Bz AMPAQGVVNK (100% 8Å match)

        Stachelhaus code match:
        :   100% (strong)

contig\_1 - Region 4 - thiopeptide

Shows the layout of the region, marking coding sequences and areas of interest. Clicking a gene will select it and show any relevant details. Clicking an area feature (e.g. a candidate cluster) will select all coding sequences within that area. Double clicking an area feature will zoom to that area. Multiple genes and area features can be selected by clicking them while holding the Ctrl key.  
More detailed help is available here.

Download region GenBank file

Download region SVG

Location: 4,441,891 - 4,468,183 nt. (total: 26,293 nt)
Show pHMM detection rules used

thiopeptide: ((YcaO or TIGR03604) and ((thio\_amide and (PF06968 or PF04055 or PF07366)) or Lant\_dehydr\_C or Lant\_dehydr\_N or PF07366 or PF06968 or PF04055) or thiostrepton)

#### Legend:

core biosynthetic genes

additional biosynthetic genes

transport-related genes

regulatory genes

other genes

resistance

reset view

zoom to selection

Gene details

Shows details of the most recently selected gene, including names, products, location, and other annotations.

Select a gene to view the details available for it

Gene overview

Thiopeptides

Gene/CDS overview

A brief tabular summary of genes/CDS features within the region.  
Filtering the table will also search biosynthetic profiles and gene function data. If enabled, the overview will then zoom to show the area covered by the filtered selection.  
Genes selected in the region drawing above will be marked in the table with an indicator to the left of the gene name.

Filter:

Automatically zoom to filtered/selected features

| Identifier | Product | Length | | Function | Sequence | | NCBI Blast | Filter details |
| --- | --- | --- | --- | --- | --- | --- | --- | --- |
|  |  | NT | AA |  | NT | AA |  |  |

No genes match the given filter

Thiopeptide predictions

Lists the possible core peptides for each biosynthetic enzyme. Predicted tail sequences are also shown, if present.   
Includes CompaRiPPson results for any available databases, with a detailed explanation here.

No core peptides found.

Thiopeptides

Core peptide predictions

Lists the possible core peptides in the region. Each core peptide lists its possible molecular weights and the scores for cleavage site prediction and RODEO. If relevant, other features, such as macrocycle and amidation, will also be listed.

No core peptides found.

contig\_1 - Region 5 - arylpolyene

Shows the layout of the region, marking coding sequences and areas of interest. Clicking a gene will select it and show any relevant details. Clicking an area feature (e.g. a candidate cluster) will select all coding sequences within that area. Double clicking an area feature will zoom to that area. Multiple genes and area features can be selected by clicking them while holding the Ctrl key.  
More detailed help is available here.

Download region GenBank file

Download region SVG

Location: 4,574,316 - 4,617,903 nt. (total: 43,588 nt)
Show pHMM detection rules used

arylpolyene: (APE\_KS1 or APE\_KS2)

#### Legend:

core biosynthetic genes

additional biosynthetic genes

transport-related genes

regulatory genes

other genes

resistance

reset view

zoom to selection

Gene details

Shows details of the most recently selected gene, including names, products, location, and other annotations.

Select a gene to view the details available for it

Gene overview

NRPS/PKS domains

Gene/CDS overview

A brief tabular summary of genes/CDS features within the region.  
Filtering the table will also search biosynthetic profiles and gene function data. If enabled, the overview will then zoom to show the area covered by the filtered selection.  
Genes selected in the region drawing above will be marked in the table with an indicator to the left of the gene name.

Filter:

Automatically zoom to filtered/selected features

| Identifier | Product | Length | | Function | Sequence | | NCBI Blast | Filter details |
| --- | --- | --- | --- | --- | --- | --- | --- | --- |
|  |  | NT | AA |  | NT | AA |  |  |

No genes match the given filter

Detailed domain annotation

Shows NRPS- and PKS-related domains for each feature that contains them. Click on each domain for more information about the domain's location, consensus monomer prediction, and other details.  
A domain glossary is available here, and an explanation of the visualisation is available here.

Selected features only

Show module domains

NRPS/PKS substrates

NRPS/PKS substrate predictions

Shows the predicted substrates for each adenylation domain and acyltransferase within genes. Each gene prediction can be expanded to view detailed predictions of each domain. Each prediction can be expanded to view the predictions by tool (and, for some tools, further expanded for extra details).

**GCF003546975\_22445**: X

:   **AMP-binding (23..285)**: X

    nrpys: (unknown)

    SVM prediction details:
    :   Predicted physicochemical class:
        :   N/A

        Large clusters prediction:
        :   N/A

        Small clusters prediction:
        :   N/A

        Single AA prediction:
        :   N/A

    Stachelhaus prediction details:
    :   Stachelhaus sequence:
        :   -GFVLAIILK

        Nearest Stachelhaus code(s):

        Stachelhaus code match:
        :   0% (weak)

If you have found antiSMASH useful, please cite us.
